# HIV antiretroviral drugs, dolutegravir, maraviroc and ritonavir-boosted atazanavir use different pathways to affect inflammation, senescence and insulin sensitivity in human coronary endothelial cells

**DOI:** 10.1371/journal.pone.0226924

**Published:** 2020-01-23

**Authors:** Martine Auclair, Anne-Claire Guénantin, Soraya Fellahi, Marie Garcia, Jacqueline Capeau

**Affiliations:** 1 Sorbonne Université, Paris, France; 2 Inserm UMR_S938, Centre de Recherche Saint-Antoine, Paris, France; 3 ICAN, Institute of Cardiometabolism and Nutrition, Paris, France; 4 Department of Biochemistry, Tenon Hospital, APHP, Paris, France; Universita degli Studi di Padova, ITALY

## Abstract

**Objectives:**

Aging HIV-infected antiretroviral-treatment (ART)-controlled patients often present cardiovascular and metabolic comorbidities. Thus, it is mandatory that life-long used ART has no cardiometabolic toxicity. Protease inhibitors have been associated with cardiometabolic risk, integrase-strand-transfer-inhibitors (INSTI) with weight gain and the CCR5 inhibitor maraviroc with improved vascular function. We have previously reported that the INSTI dolutegravir and maraviroc improved, and ritonavir-boosted atazanavir(atazanavir/r) worsened, inflammation and senescence in human coronary artery endothelial cells (HCAEC)s from adult controls. Here, we analyzed the pathways involved in the drugs’ effects on inflammation, senescence and also insulin resistance.

**Methods:**

We analyzed the involvement of the anti-inflammatory SIRT-1 pathway in HCAECs. Then, we performed a transcriptomic analysis of the effect of dolutegravir, maraviroc and atazanavir/r and used siRNA-silencing to address ubiquitin-specific-peptidase-18 (USP18) involvement into ART effects.

**Results:**

Dolutegravir reduced inflammation by decreasing NFκB activation and IL-6/IL-8/sICAM-1/sVCAM-1 secretion, as did maraviroc with a milder effect. However, when SIRT-1 was inhibited by splitomicin, the drugs anti-inflammatory effects were maintained, indicating that they were SIRT-1-independant.

From the transcriptomic analysis we selected USP18, previously shown to decrease inflammation and insulin-resistance. USP18-silencing enhanced basal inflammation and senescence. Maraviroc still inhibited NFκB activation, cytokine/adhesion molecules secretion and senescence but the effects of dolutegravir and atazanavir/r were lost, suggesting that they involved USP18. Otherwise, in HCAECs, dolutegravir improved and atazanavir/r worsened insulin resistance while maraviroc had no effect. In USP18-silenced cells, basal insulin resistance was increased, but dolutegravir and atazanavir/r kept their effect on insulin sensitivity, indicating that USP18 was dispensable.

**Conclusion:**

USP18 reduced basal inflammation, senescence and insulin resistance in coronary endothelial cells. Dolutegravir and atazanavir/r, but not maraviroc, exerted opposite effects on inflammation and senescence that involved USP18. Otherwise, dolutegravir improved and atazanavir/r worsened insulin resistance independently of USP18. Thus, in endothelial cells, dolutegravir and atazanavir/r oppositely affected pathways leading to inflammation, senescence and insulin resistance.

## 1. Introduction

Aging persons living with HIV, well-controlled by antiretroviral treatment(ART), present a high prevalence of age-related cardiovascular and metabolic comorbidities [1–4], higher than the prevalence observed in non-infected individuals with similar risk factors [3]. Therefore, in these patients, it is mandatory to favor ART with minimal metabolic and cardiovascular toxicity.

Some contemporary used protease inhibitors (PI) have been associated with an increased cardiovascular risk [5–7], in part related to the boosting concentration of ritonavir, which leads to raised LDL-cholesterol and triglycerides levels. This has been clearly shown for ritonavir-boosted lopinavir [5, 8], and ritonavir-boosted darunavir [9]. Ritonavir-boosted atazanavir (ATV/r) has been associated with a lower cardiovascular risk than ritonavir-boosted darunavir [9]. This could be related to the ability of ATV/r to increase bilirubin levels, because bilirubin has been related to cardio-protective anti-oxidant effects [10].

Integrase strand transfer inhibitors (INSTI) have been initially considered as lipid- and metabolic-friendly. Because they exert potent anti-viral activities, they are recommended at present for treatment initiation in ART-naïve patients and also for switch strategies in ART-controlled patients with comorbidities. A decreased level of proatherogenic lipids has been consistently reported in patients switched to INSTI [4, 11–14]. As well, no increased cardiovascular risk has been observed [15] and, even, recently, a lower cardiovascular disease risk has been associated with INSTI versus other regimens after a median follow-up of 18 months in more than 20 000 ART initiators[16].

However, recently, several reports revealed treatment with that some INSTIs resulted in weight gain, both in ART-initiated and ART-experienced patients switched off PIs to INSTI [12, 13, 17–22]. This may represent an undesirable effect placing patients at higher risk for cardiovascular and metabolic complications on the long term. Discrepant results were reported regarding the impact of INSTI on insulin sensitivity, some studies arguing for an improvement, and others for no change or even a worsening of insulin resistance [4, 12, 23–25].

Regarding maraviroc (MVC), its ability to modulate atherosclerotic progression and to improve endothelial function was shown in two small studies, this improvement being associated, in one of them, with decreased inflammatory markers [26, 27].

We have previously reported that DTG, raltegravir, MVC, ATV/r and ritonavir-boosted darunavir differentially affected endothelial cells [28]. DTG exerted anti-inflammatory effects and reduced senescence, as did MVC to a lesser extent, while we observed the reverse with the two PIs. The aim of the present paper was to further decipher the cellular pathways leading to inflammation, senescence and insulin sensitivity in HCAECs and which were altered by MVC, DTG and ATV/r. At first, we tested the possibility that the SIRT-1 pathway, which activation results in decreased inflammation and senescence in endothelial cells, could explain the effect of the antiretroviral molecules. We could not confirm this hypothesis. Thus, we used a non-targeted transcriptomic approach and identified USP18 as a probable signaling step in the effect of DTG and ATV/r, but not MVC, on inflammation and senescence. Moreover, DTG and ATV/r, but not MVC, altered insulin sensitivity and their effects were not dependent on USP18. Taken as whole, DTG and ATV/r appeared to oppositely alter the same pathways different from those used by MVC.

## 2. Material and methods

### 2.1 Cell culture and cell treatments

We used Human primary Coronary Artery Endothelial Cells (HCAEC, PromoCell, Heidelberg, Germany) isolated from the coronary arteries from adult donors. To perform the entire set of experiments we used coronary endothelial cells issued from four different donors: two male and two female subjects, devoid of cardiovascular morbidity, non-obese, non-diabetic, aged 27, 35, 40 and 57 years old, two Caucasian, one Hispanic and one Hispanic/Black. We used, for all cultures, the endothelial cell growth medium from Promocell (C22010) supplemented with 5% fetal calf serum. Cell were grown in 6-well dishes from passages 2–8 and used when confluent.

We verified by Western blot (antibody MEM-111, ref ab2213 from Abcam, Cambridge, UK, dilution 1/1000) that the CCR5 receptor was expressed on endothelial cells, as previously shown[29, 30]. We confirmed its expression in each coronary cell line. Moreover, when cells were incubated for 15 days with MVC, DTG and ATV/r, the mean level of CCR5 was not modified (one-way Anova with Dunnett’s multiple comparisons tests NS, S1 Fig).

ATV, ritonavir and MVC were purchased from Santa Cruz Biotechnology, Inc. (Santa Cruz, CA, USA), DTG from Medchem Express (Princeton, NJ, USA). HCAEC were treated for 15 days with DMSO 0.1% without (control) or with the drugs dissolved in DMSO at Cmax plasma concentrations according to the literature [31] or to the Liverpool HIV-interactions website (www.hiv-druginteractions.org): MVC 0.9 μg/ml, DTG 3.7 μg/ml, ATV/r 5.2 and 0.94 μg/ml. Three independent experiments were performed for each protocol. The results are all expressed as the mean values of the 3 experiments related to the % of the control established at 100%.

### 2.2 SIRT-1 pathway

We analyzed the effect of a SIRT-1 activator SRT1720 [32]. Regarding dose-response experiments [33], at first, we evaluated concentrations between 0 and 10 μM for 48h. However, since the higher concentrations (2.5–10 μM) induced cell toxicity, we further analyzed the effect of SRT1720 at concentrations 0.5, 1 and 2 μM. The absence of toxicity was evaluated by visual inspection and by the verification that the total protein amount in each dish was equivalent to that of the control at the end of the experiment. The efficacy of SRT1720 was evaluated by its ability to inhibit NFκB. We determined that the concentration of 2 μM was optimal to induce SIRT-1 and inhibit NFκB. We also evaluated the effect of a SRTI-1 inhibitor, splitomicin [34]. We tested splitomicin at concentrations ranging from 0 to 500 μM according to the literature[35]. We discarded the 500 μM concentration which was toxic and evaluated the ability of splitomicin to induce NFκB at concentrations of 50, 100, 200 μM. The dose-response evaluation allowed to determine that the concentration of 100 μM was optimal to decrease SIRT-1 and to activate NFκB. Thereafter, these concentrations of SRT-1720 and splitomicin were used in all the experiments performed. The cells were cultured during 15 days with/without the antiretroviral molecules, SRT1720 or splitomycin being added for the last 3 days.

### 2.3 Western blotting

Whole cell lysates were subjected to sodium dodecyl sulfate/polyacrylamide gel electrophoresis and Western blotting. We used antibodies against USP18 (sc-374064), AKT (sc-8312), phospho-AKT (sc-7985R), phosphotyrosine (sc-7020) from Santa Cruz at dilution 1/500; against NFκB p65/RelA (8242S), phospho(S536)-NFκB p65/RelA (3033S), insulin receptor beta-subunit (3025), JNK (9252), phospho-JNK (9251S), TAK1 (45055) and phospho-TAK-1(4531) from Cell Signalling Technology, Inc. (Danvers, MA, USA) at dilution 1/1000; against p16 (10883-1-AP) and p21 (10355-1-AP) from Proteintech (Proteintech Europe, Manchester, UK) or from BD Biosciences (San Jose, CA, USA) at dilution 1/1000; against ICAM-1 (ab2213) and VCAM-1 (ab134047) from Abcam (Abcam, Cambridge, UK) at dilution 1/1000; against tubulin, used as an index of the cellular protein content, from Sigma-Aldrich (Saint Louis, MO, USA) at dilution 1/1000. For antibodies against SIRT1 we used at first antibodies from Santa Cruz (sc15404) at dilution 1/500 then from Abcam (ab32441) at dilution 1/1000.

The activity of NFκB was measured by the ratio of the level of phosphorylation of the p65/RelA protein to the total level of p65/RelA, as measured by Western blot[36].

### 2.4 Secretion of pro-inflammatory cytokines and adhesion molecules

The secretion of interleukin-6 (IL-6), interleukin-8 (IL-8), soluble intracellular cell adhesion molecule-1 (sICAM-1) and soluble vascular cell adhesion molecule-1 (sVCAM-1) in 24-h culture supernatants was evaluated by ELISA assays (R&D quantikine HS600C and HS800 for human IL-6HS and IL-8HS, DVC00 and DCD540 for human VCAM-1 and ICAM-1) or using the ELLA system (Protein Simple, Simple Plex Cartridge for IL-6 and Simple Plex Cartridge for IL-8, Bio Techne, Minneapolis, MN, USA). Cells were incubated for 14 days with/without the drugs. The culture medium was replaced for the last 24 hours by a medium without foetal calf serum with/without the drugs and collected after 24 hours to measure the level of secreted cytokines and adhesion factors.

### 2.5 RT-PCR

Cells were incubated with drugs during 15 days. Total mRNA was extracted followed by reverse transcription (capacity cDNA reverse transcription kit, #4368814, Life Technologies). Quantitative PCR was performed with LightCycler 480 using LightCycler 480 SYBR Green I Master mix (#04887352001, Roche Diagnostics, Meylan, France).

The primers were designed by using the “Assay design center” of Roche.

https://lifescience.roche.com/en_gb/brands/universal-probe-library.html and ordered to Invitrogen (Thermo Fisher Scientific, Waltham, MA, USA). The primer sequence for USP18 was: hUSP18 S: TCC CGA CGT GGA ACT CAG h USP18 AS: CAG GCA CGA TGG AAT CTC TC

We used two reference genes: HPRT-1 and PPIA.

### 2.6 Transcriptomics

RNA-quality was evaluated according to good practice of Genom’IC facility (Institut Cochin, Paris, France) by Agilent Bioanalyzer 2100 before being spotted on human Gene 2.0 ST arrays. After an RMA (Robust Multi-array Average) with Bioconductor, differentiated gene analysis of data was performed by One-Way Repeated Measure ANOVA (paired). Enrichment analysis of this gene set (p<0.05 over treatment vs. control) was carried out with IPA software (Ingenuity pathway analysis, www.ingenuity.com).

### 2.7 Gene silencing

We used the protocol from Santa-Cruz biotechnologies for siRNA-mediated inhibition of gene expression. The specific USP18 siRNA and the scramble siRNA (SC-37007), which contained a scrambled sequence that will not lead to the specific degradation of any known cellular mRNA, were ordered from Santa Cruz. The transfection was performed in the presence of the transfection medium (SC-36868 and SC-29528) in 80% confluent endothelial cells in 6-well dishes, for 6h at 37°C.

### 2.8 Senescence markers

We evaluated the protein level of two cell-cycle inhibitors associated with cellular senescence, p16^INK4^ and p21^WAF1^ by Western blot as previously described[28].

### 2.9 Insulin sensitivity

The basal level of insulin sensitivity was evaluated by the production of nitric oxide (NO) as previously described[31]. NO production was assessed with the cell-permeant NO indicator 4-amino-5-methylamino-2′,7′-difluorofluorescein diacetate (DAF-FM; D23844; Molecular Probes, Life Technologies, Carlsbad, CA, USA). Cells were cultured in 96-well plates, washed and incubated with DAF-FM (12.5 μmol/l) or Hoechst 33258 (0.01 mg/ml) in DMEM without fetal bovine serum for 30 min at 37°C in the dark. Quantification was performed with a plate fluorescence reader (Infinite M200; Tecan-France, Trappes, France) at 515 nm (DAF-FM) and 460 nm (Hoechst 33258).

We evaluated the cell response to insulin at two key steps of the insulin signaling pathways, tyrosine phosphorylation of the insulin receptor-β subunit (level of tyrosine-phosphorylated β-subunit reported to the level of total insulin receptor β-subunit), and activation of AKT (level of phospho-AKT reported to the level of total AKT) as previously published [37, 38]. In brief, cell grown in 6-well dishes were cultured to confluence, then depleted from fetal bovine serum for 16 h, and stimulated for 15 min with insulin at 100 nM. Western blotting analysis used antibodies as indicated above.

### 2.10 Statistics

Comparison of the different points with the cells incubated with the different drugs dissolved in DMSO to the control value being set at 100% was performed at first by using one-way ANOVA (or two-way ANOVA for experiments with USP18 silencing) with Dunnett’s multiple comparisons tests, by comparing each experimental point to the relevant control with the Prism software. If ANOVA indicated a significant difference, we further evaluated the differences between the respective control and each drug treatment by using the Student T-test with the Welch’s correction with the Prism software. The results regarding significant differences obtained with one-way ANOVA with Dunnett’s multiple comparison tests and with Student T-test with Welch’s correction were globally similar.

## 3. Results

### 3.1 Effect of the antiretrovirals and of SIRT-1 activation or inhibition on the SIRT-1 level and on inflammation

#### 3.1.1 Effect of antiretrovirals and of SRT1720 or splitomycin on the protein level of SIRT-1

**Fig 1:**
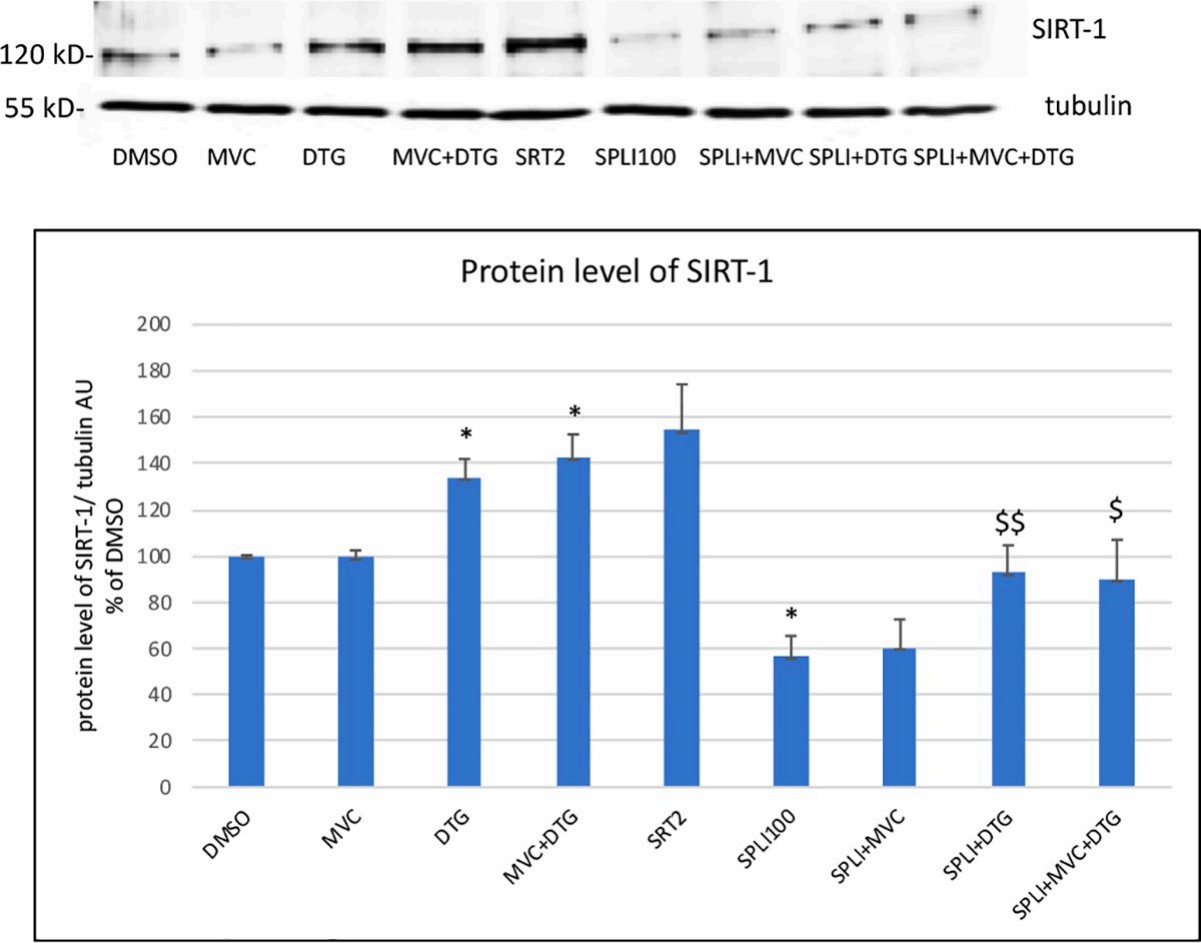
Effect of the antiretrovirals and of the SIRT-1 activator (SRT1720) and inhibitor (splitomycin) on the protein level of SIRT-1. A representative blot of SIRT-1 and tubulin as a loading control is shown. The histograms represent the mean+/-SEM value as compared to the DMSO control set at 100% of 4 independent experiments. The comparisons used Welch’s correction of Student T-test. SRT2: SRT1720 2 μM, Spli100: splitomicin 100μM * p<0.05 versus control (DMSO) $ p<0.05, $ $ p<0.01$ $ versus Spli100.

We observed that DTG and DTG+MVC increased SIRT-1 respectively by 34% and 43% while MVC had no effect (Fig 1). The SIRT-1 activator, SRT1720, tended to increase the SIRT-1 level (+54%, p = 0.07) while the inhibitor splitomycin decreased this level by 44%. However, in the presence of splitomicin, DTG alone or associated with MVC kept its ability to increase the level of SIRT-1 (+64% and +61% as compared to splitomicin alone).

#### 3.1.2 Effect of antiretrovirals and of SRT1720 or splitomycin on NFκB activation and secretion of cytokines and adhesion molecules

**Fig 2:**
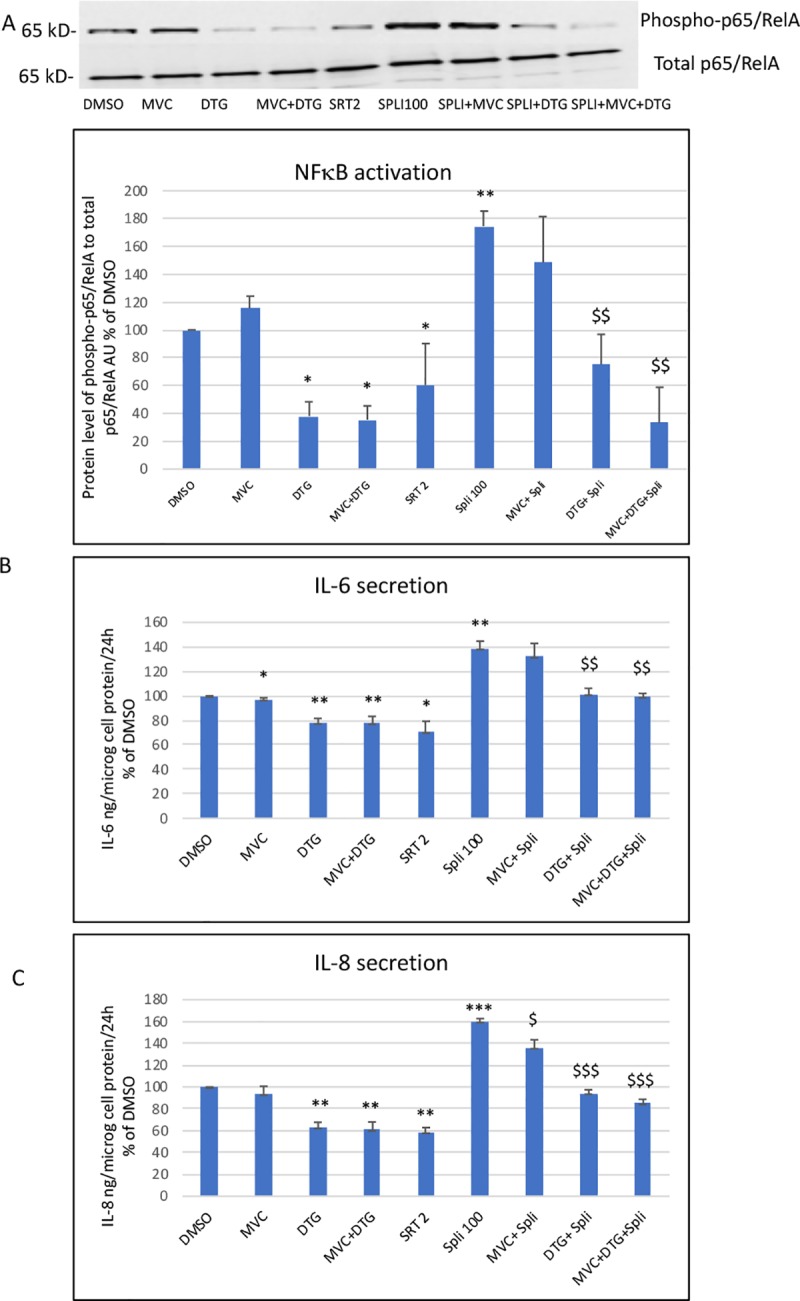
**Effect of the antiretrovirals and of the SIRT-1 activator (SRT1720) and inhibitor (splitomycin) on NFκB activation (A) and secretion of IL-6 (B) and IL-8 (C).** A representative blot of phospho-p65/RelA and total p65/RelA is shown. The level of NFκB activation was evaluated by the ratio of phospho-p65/RelA to total p65/RelA. IL-6 and IL-8 secretion was evaluated as the level of IL-6 or IL-8 produced in 24 h in the culture medium and related to the total cellular protein content. The histograms represent the mean+/-SEM value as compared to the DMSO control set at 100% of 4 independent experiments. SRT2: SRT1720 2 μM, Spli100: splitomicin 100μM The comparisons used Welch’s correction of Student T-test. * p<0.05, ** p<0.01 versus control (DMSO) $ $ p< 0.01, $ $ $ p< 0.001 versus spli100.

As expected, DTG, DTG+MVC and SRT1720 inhibited NFκB while splitomycin activated it (Fig 2A). In the presence of splitomicin, DTG and the association DTG+MVC were still able to decrease NFκB activation. MVC had no effect (Fig 2A).

DTG and DTG+MVC significantly decreased the secretion of IL-6 and IL-8 by endothelial cells and this was also the case for SRT1720. Conversely, splitomycin increased the secretion of the two cytokines. This effect was reversed in part by DTG and DTG+MVC. MVC has a mild inhibitory effect on IL-6 and IL-8 secretion (Fig 2B and 2C). We obtained similar results for the secretion of sICAM-1 and sVCAM-1. Treatment with DTG and DTG+MVC resulted in decreased secretion (by 46% for ICAM-1 and 43–49% for VCAM-1) of the two adhesion molecules. Splitomycin increased ICAM-1 (by 44%) and VCAM-1 (by 52%) secretion. DTG and DTG+MVC were still able to decrease ICAM-1 (by 20–22%) and VCAM-1 (by 44–45%) secretion in splitomicin-treated cells. MVC had no effect.

Thus, DTG, and to a lesser extent MVC, reduced NFκB activation, resulting in decreased secretion of IL-6, IL-8, sVCAM-1 and sICAM-1. Their effects were not mediated by SIRT-1.

### 3.2 Transcriptomic analysis of the effect of antiretrovirals in endothelial cells

**Fig 3:**
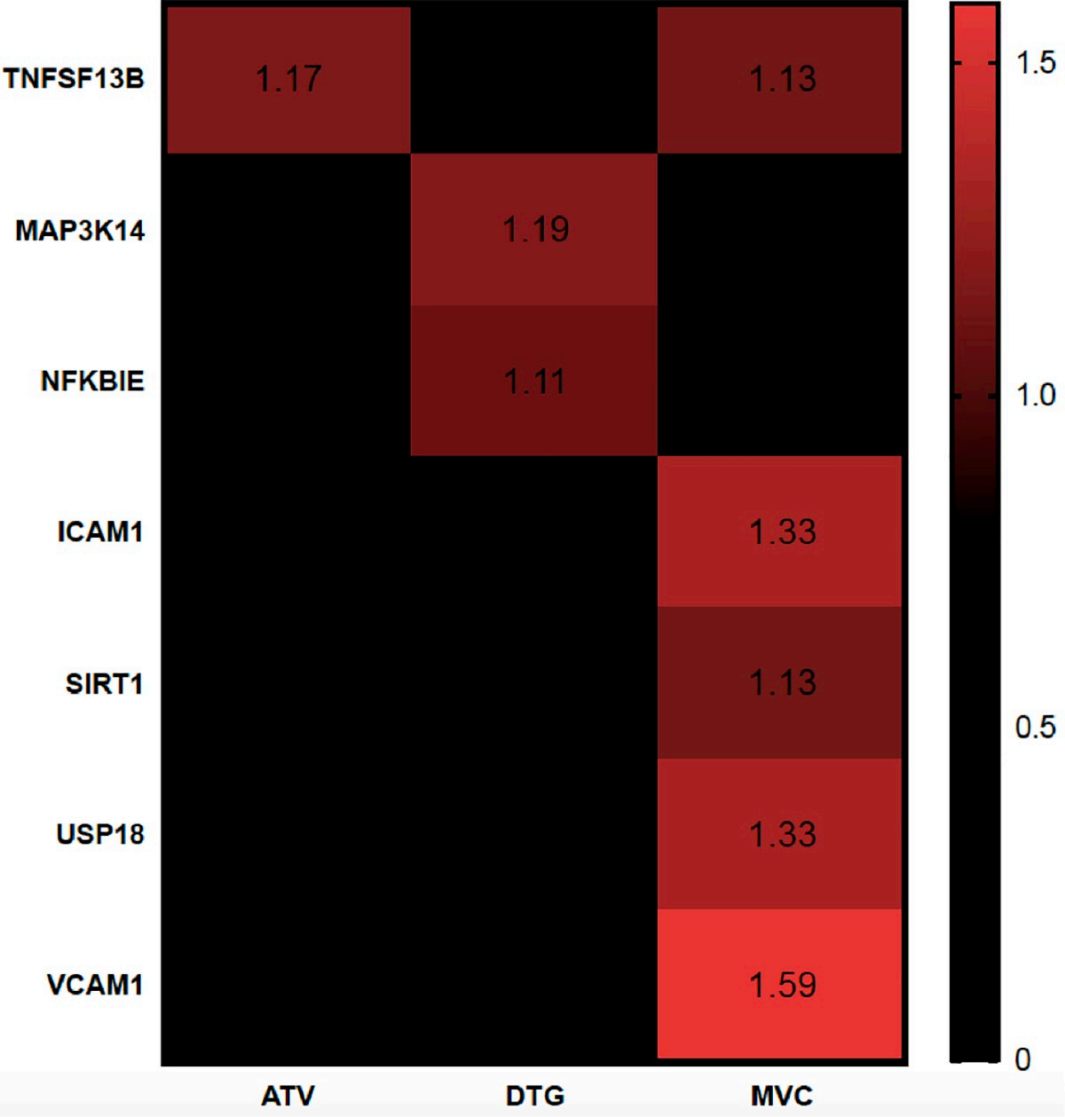
Heatmap representing identified genes of interest which level was affected by antiretrovirals. Values are presented as follows: fold change in gene expression upregulated vs control (red). Black cases represent non-significative variation of genes vs control. TNFSF13B: tumor necrosis factor ligand superfamily member 13 or B-cell activating factor (BAFF); MAP3K14: mitogen activated protein kinase kinase kinase 14; NFKBIE: NFκB inhibitor epsilon.

To further analyze the pathways that were modified by a treatment with DTG, MVC and ATV/r we performed a global untargeted transcriptomic analysis, comparing the mRNA profile obtained in the presence of each drug to the control condition. We identified several genes which expression was differentially affected by the drugs (Fig 3). Among these different genes, we selected USP-18 since it has been involved in interferon signaling but also in insulin sensitivity and inflammation[39–41]. The results of the transcriptomic analysis can be accessed through the link *https*:*//www*.*ncbi*.*nlm*.*nih*.*gov/geo/query/acc*.*cgi*?*acc=GSE137247*

**Fig 4:**
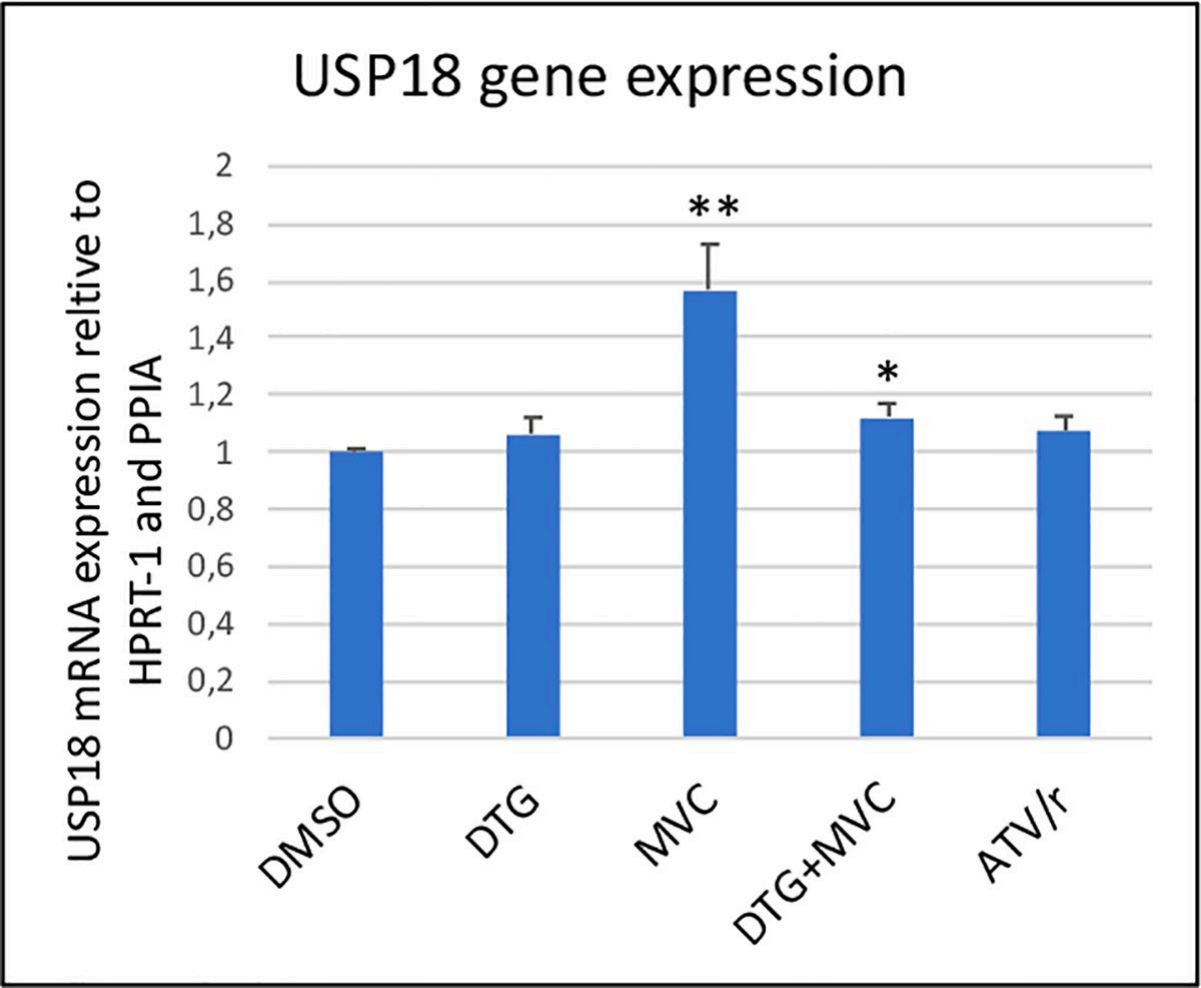
Gene expression of USP18 in the presence of the antiretrovirals. USP18 mRNA level was analyzed by RT-PCR. Two housekeeping genes were used, HPRT-1 and PPIA. The histograms represent the mean+/-SEM value as compared to the DMSO control set at 100% of 3 independent experiments. The comparisons used Welch’s correction of Student T-test. * p<0.05, ** p<0.01 versus control (DMSO).

RT-PCR analysis confirmed that that the mRNA level of USP18 was increased by MVC by 57% but not by DTG or ATV/r. (Fig 4)

### 3.3 Silencing of USP18

#### 3.3.1 Effect on the protein level of USP18

**Fig 5:**
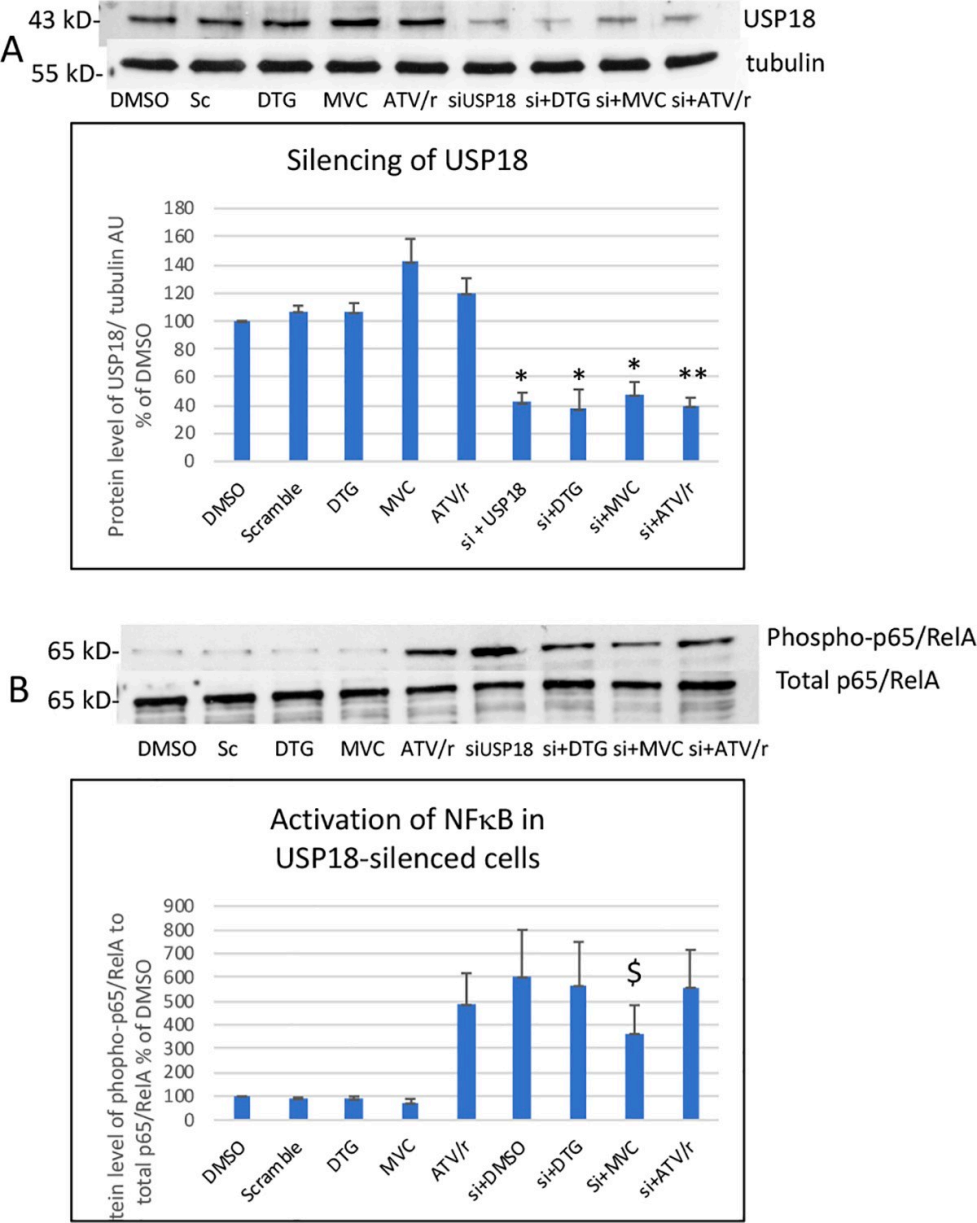
**Effect of USP18 silencing on the level of the USP18 protein (A) and on the ability of DTG, MVC and ATV/r to modulate NFκB activation (B)** A representative Western blot of USP18 and tubulin as a loading control is shown in A and of phospho-p65/RelA and total p65/RelA to evaluate NFκB activation in B. The histograms represent the mean+/-SEM value as compared to the DMSO control set at 100% of 3 independent experiments. Si-DMSO: control silenced for USP18, Si-DTG: DTG-treated cells silenced for USP18, Si-MVC: MVC-treated cells silenced for USP18, Si-ATV/r: ATV/r-treated cells silenced for USP18 The comparisons used Welch’s correction of Student T-test. * p< 0.05 ** p<0.01 versus control (DMSO) $ p< 0.05 versus Si-DMSO.

We analyzed the protein level of USP18 in endothelial cells incubated with the specific USP18 siRNA or with a scramble siRNA taken as a control (Fig 5A). MVC tended to increase USP18 level (p = 0.12) in accordance with increased mRNA level. USP18 silencing resulted in a 61–67% reduction in the USP18 protein level as compared to the level observed in cells incubated with the scramble siRNA (Fig 5A).

#### 3.3.2 Activation of NFκB and secretion of cytokines and adhesion molecules

**Fig 6:**
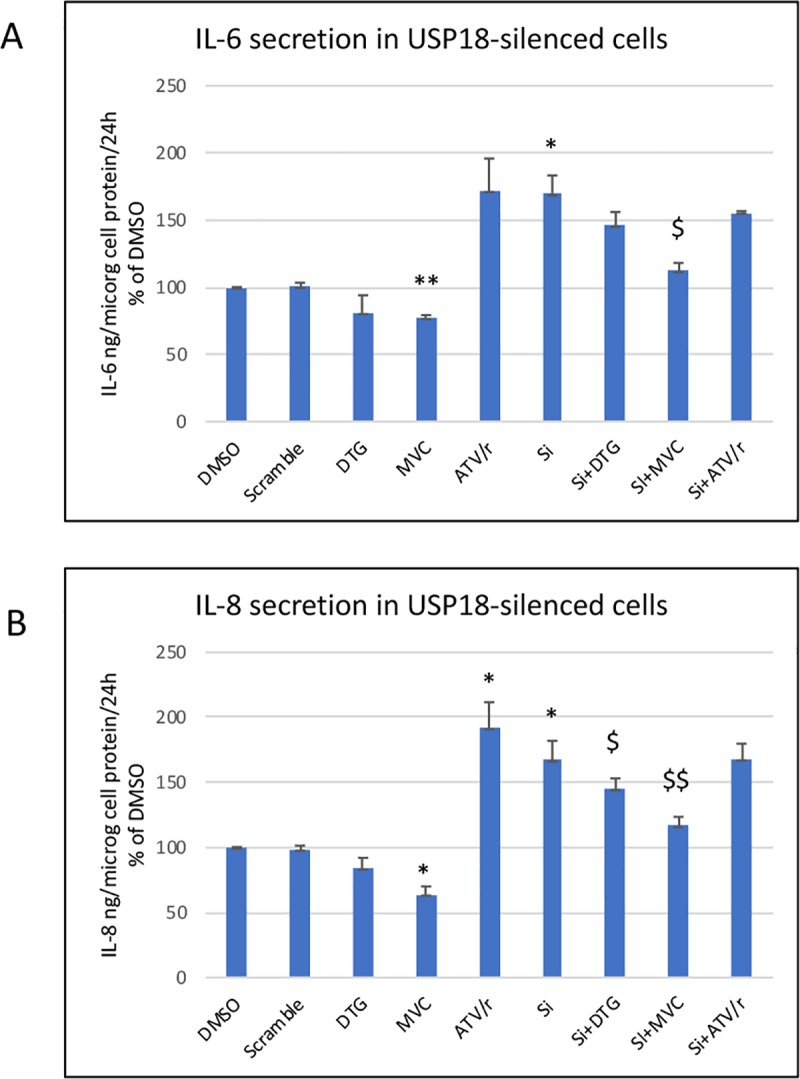
**Effect of USP18 silencing on the level of the IL-6 (A) and IL-8 secretion (B)** The level of IL-6 and IL-8 was evaluated in the last 24-hour culture medium and reported to the total cellular protein level in each well. The histograms represent the mean+/-SEM value as compared to the DMSO control set at 100% of 4 independent experiments. Si-DMSO: control silenced for USP18, Si-DTG: DTG-treated cells silenced for USP18, Si-MVC: MVC-treated cells silenced for USP18, Si-ATV/r: ATV/r-treated cells silenced for USP18 The comparisons used Welch’s correction of Student T-test. * p< 0.05, ** p<0.01 versus control (DMSO) $ p< 0.05, $ $ p<0.01versus Si-DMSO.

Silencing of USP18 resulted in a 6-fold increased basal level of NFκB activation. MVC decreased by 49% this level of activation while DTG and ATV/r had lost their effect (Fig 5B). As well, in USP18-silenced cells the basal level of IL-6 secretion was 69% higher than in cells treated with the scramble construction (Fig 6A). In USP-silenced cells, MVC decreased IL-6 secretion by 33% while DTG and ATV/r had no significant effect. Accordingly, IL-8 secretion was increased by 67% when USP18 was silenced, MVC reduced this secretion in USP-silenced cells by 30% while DTG has a minor effect (13% decrease) and atazanavir no effect (Fig 6B).

**Fig 7:**
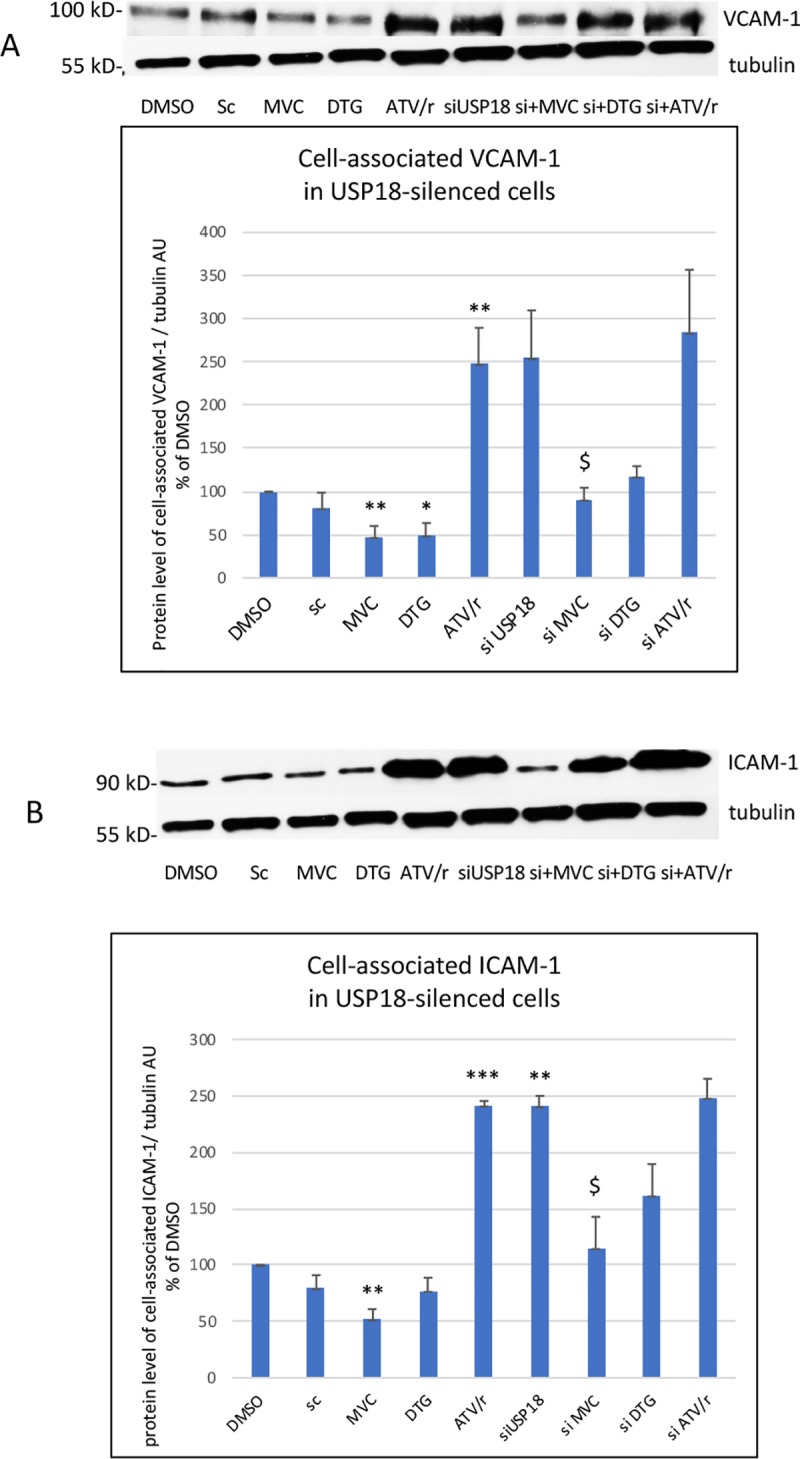
Effect of USP18-silencing on the level of cell-associated VCAM-1 and ICAM-1. A representative Western blot of VCAM-1 and tubulin as a loading control is shown in A and of ICAM-1 and tubulin in B. The histograms represent the mean+/-SEM value as compared to the DMSO control set at 100% of 3 independent experiments. Si-DMSO: control silenced for USP18, Si-DTG: DTG-treated cells silenced for USP18, Si-MVC: MVC-treated cells silenced for USP18, Si-ATV/r: ATV/r-treated cells silenced for USP18 The comparisons used Welch’s correction of Student T-test. * p< 0.05, ** p<0.01, ***p<0.001 versus control (DMSO) $ p< 0.05, versus Si-DMSO.

We observed similar results for sICAM-1 and sVCAM-1. Moreover, we evaluated the cell-associated level of ICAM-1 and VCAM-1. This level was reduced by MVC and DTG and increased by ATV/r (Fig 7). Silencing of USP18 resulted in an increased level of cell-associated ICAM-1 and VCAM-1. In silenced cells, the effects of MVC were maintained but those of DTG and ATV/r were reduced or lost.

Thus, MVC increased the mRNA but not the protein level of USP18. Its inhibitory effect on NFκB activation and cytokine/adhesion molecules secretion was enhanced in USP18-silenced cells. Conversely, the effect of DTG and ATV/r was lost or reduced in USP18-silenced cells indicating that USP18 was required for the anti- and pro-inflammatory effects of DTG and ATV/r on NFκB activation and cytokine/adhesion molecules secretion.

#### 3.3.3 Effect of USP-silencing on the level of cell-cycle inhibitors

**Fig 8:**
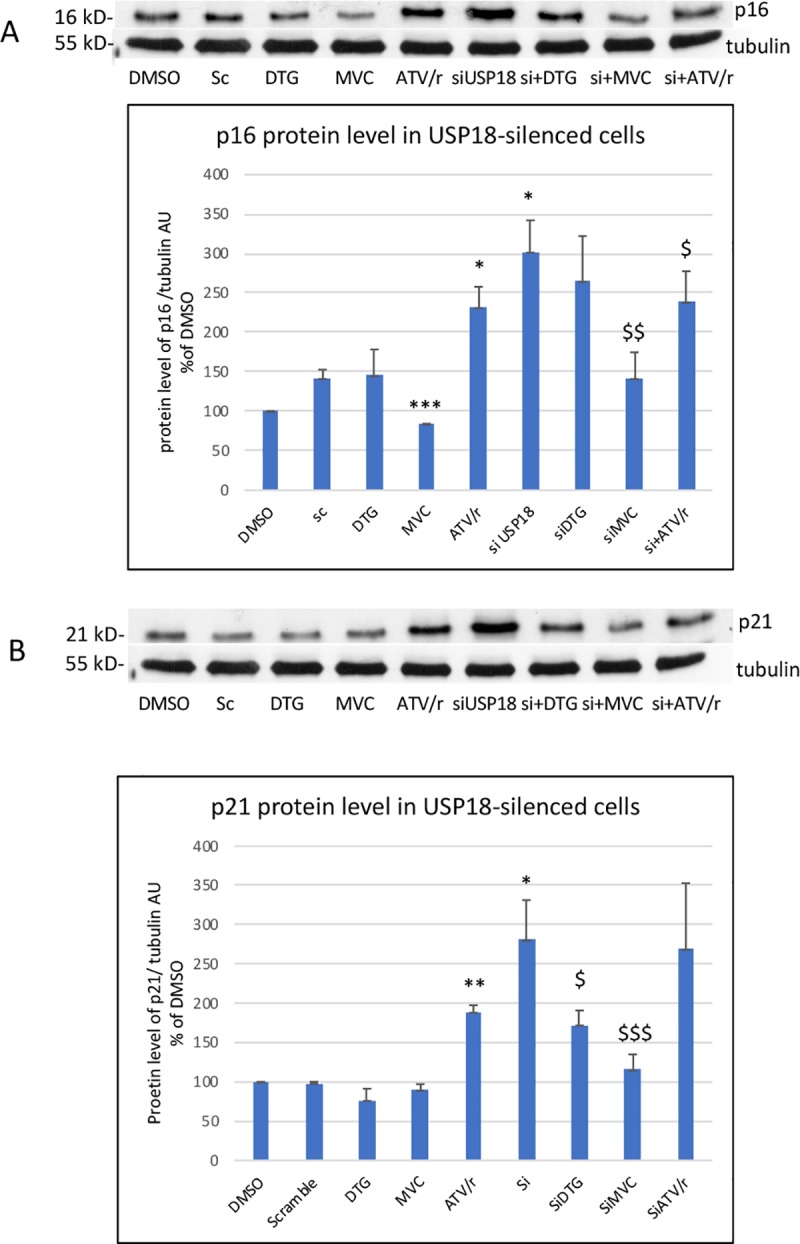
Effect of USP18 silencing on the ability of DTG, MVC and ATV/r to modulate the protein level of p16 and p21. Representative Western blots are shown of p16 and tubulin as a loading control in A and of p21 and tubulin in B. The histograms represent the mean+/-SEM value as compared to the DMSO control set at 100% of 3 independent experiments. Si-DMSO: control silenced for USP18, Si-DTG: DTG-treated cells silenced for USP18, Si-MVC: MVC-treated cells silenced for USP18, Si-ATV/r: ATV/r-treated cells silenced for USP18. The comparisons used Welch’s correction of Student T-test. * p<0.05, *** p<0.001 versus control (DMSO) $ p< 0.05, $ $ p<0.01 versus Si-USP18 (Si-DMSO).

We have previously shown that antiretrovirals were able to modulate the basal level of endothelial cell senescence[28]. To go further, we analyzed whether this parameter was altered in USP18-silenced cells by measuring the protein level of the two cell-cycle inhibitors strongly linked to the senescence phenotype, p16^INK4^ and p21^WAF1^. We observed that their level was increased in silenced cells (Fig 8) and that MVC was still able to decrease their level. Conversely, the ability of DTG and ATV/r to respectively decrease and increase the level of the senescence proteins was lost or minored, suggesting that USP18 was involved in the effects of DTG and ATV/r on senescence but not in the effect of MVC.

#### 3.3.4 Insulin sensitivity

**Fig 9:**
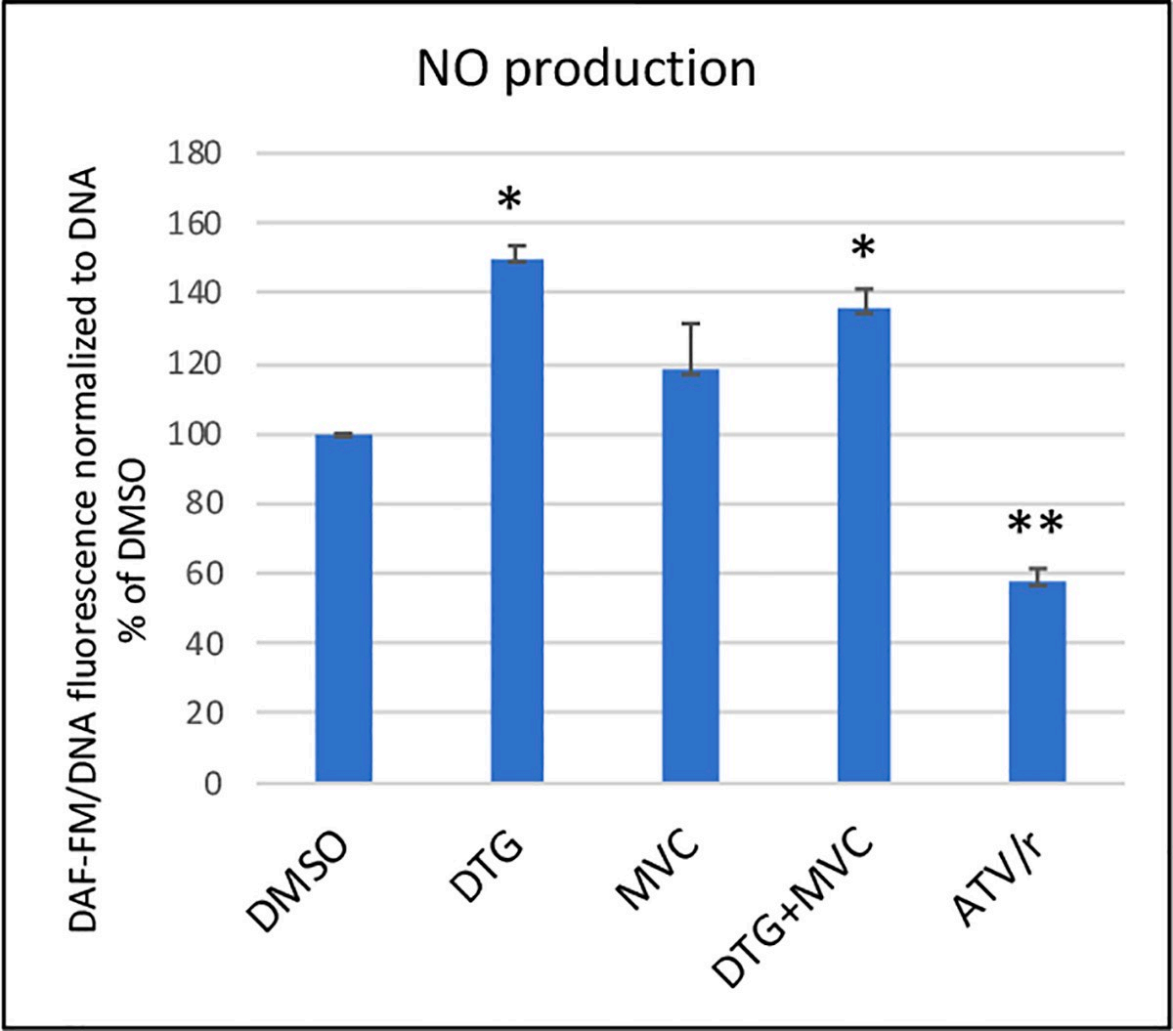
Ability of antiretrovirals to modulate nitric oxide secretion by endothelial cells. NO production was assessed with the cell-permeant NO indicator 4-amino-5-methylamino-2′,7′-difluorofluorescein diacetate (DAF-FM) normalized to DNA (Hoechst 33258 fluorescence). The histograms represent the mean+/-SEM value as compared to the DMSO control set at 100% of 3 independent experiments. The comparisons used Welch’s correction of Student T-test. * p< 0.05, ** p<0.01 versus control (DMSO).

Then, we addressed the ability of the drugs to modify insulin sensitivity. In the basal state, we evaluated the production, in the culture medium, of nitric oxide, reflecting the state of insulin sensitivity[42]. We observed that DTG increased, while ATV/r reduced, NO production, indicating respectively enhanced and decreased basal insulin sensitivity. MVC did not modify it (Fig 9).

**Fig 10:**
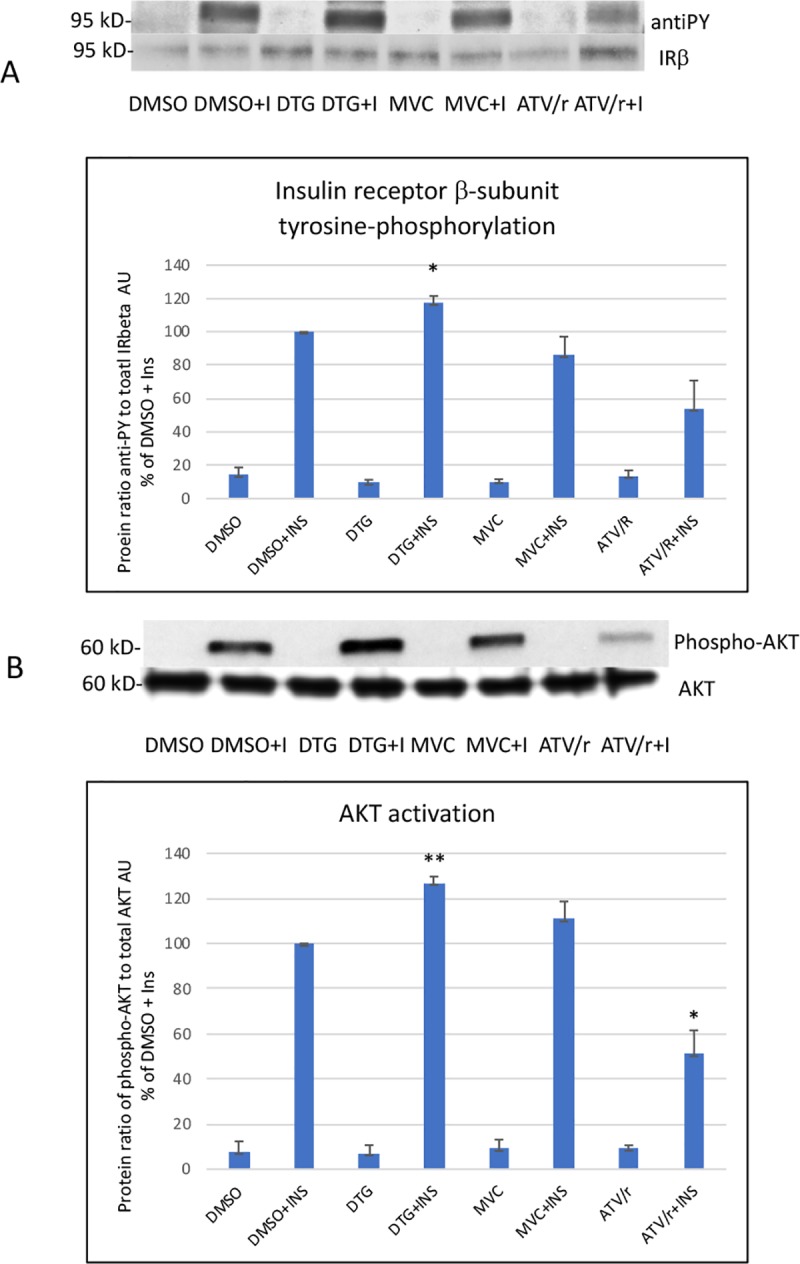
**Effect of DTG, MVC and ATV/r on the ability of insulin to activate insulin receptor** **β****-subunit tyrosine phosphorylation (A) and AKT (B).** A representative Western blot of the level of tyrosine phosphorylation and of the total level of insulin receptor β-subunit is shown in A and of phospho-AKT and total AKT in B. Insulin receptor activation was analyzed by the ratio of tyrosine phosphorylation to the total level of the insulin receptor β-subunit and AKT activation by the ratio of phospho-AKT to total AKT The histograms represent the mean+/-SEM value as compared to the DMSO control set at 100% of 3 independent experiments. Anti-PY: anti-phospho-tyrosine, IRβ: insulin receptor beta-subunit, I or INS: insulin The comparisons used Welch’s correction of Student T-test. * p<0.05, ** p<0.01 versus control (DMSO+INS).

To go further, we analyzed cell response to insulin by measuring the level of tyrosine phosphorylation of the insulin receptor β-subunit and the level of phosphorylation of AKT (ratio of phospho-AKT to total AKT), involved in the metabolic insulin signaling pathway. DTG increased and ATV/r decreased (or tended to decrease p = 0.1) insulin receptor and AKT activation while MVC had no effect (Fig 10A and 10B). The total protein level of the insulin receptor β-subunit reported to that of tubulin did not differ in cells treated or not with the drugs.

**Fig 11:**
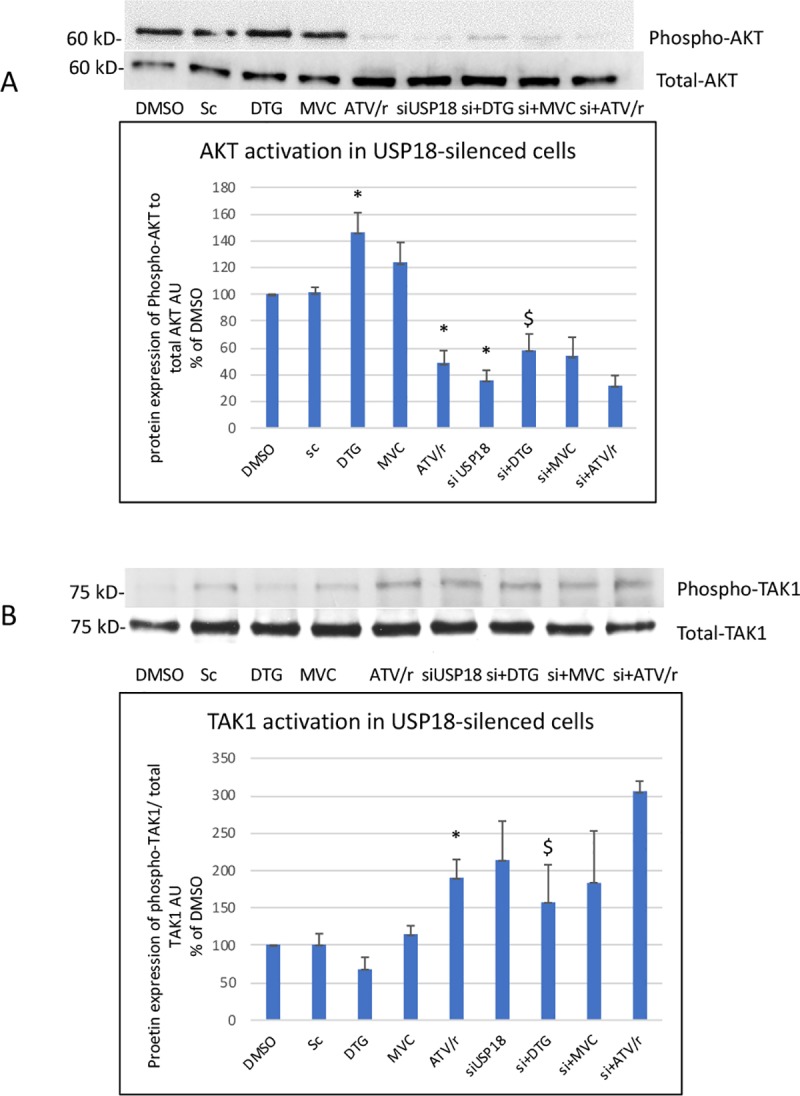
**Effect of USP18 silencing on the ability of DTG, MVC and ATV/r to modulate basal activity of AKT (A) and TAK-1 (B) involved in insulin signaling/resistance.** Representative Western blots of phospho-AKT and total AKT in A and phospho-TAK1 and total TAK-1 in B are shown. The histograms represent the mean+/-SEM value as compared to the DMSO control set at 100% of 3 independent experiments. Si-DMSO: control silenced for USP18, Si-DTG: DTG-treated cells silenced for USP18, Si-MVC: MVC-treated cells silenced for USP18, Si-ATV/r: ATV/r-treated cells silenced for USP18. The comparisons used Welch’s correction of Student T-test. * p<0.05, ** p<0.01 versus control (DMSO).

We then evaluated the level of insulin resistance in USP18-silenced cells by addressing AKT activation. In cells treated with the scramble construction, we confirmed that DTG increased and ATV/r decreased insulin sensitivity (Fig 11A). USP18 silencing increased basal insulin resistance (decreased AKT activation). However, the effect of DTG was maintained in silenced cells, indicating that it did not involve USP18 (Fig 11A).

**Fig 12:**
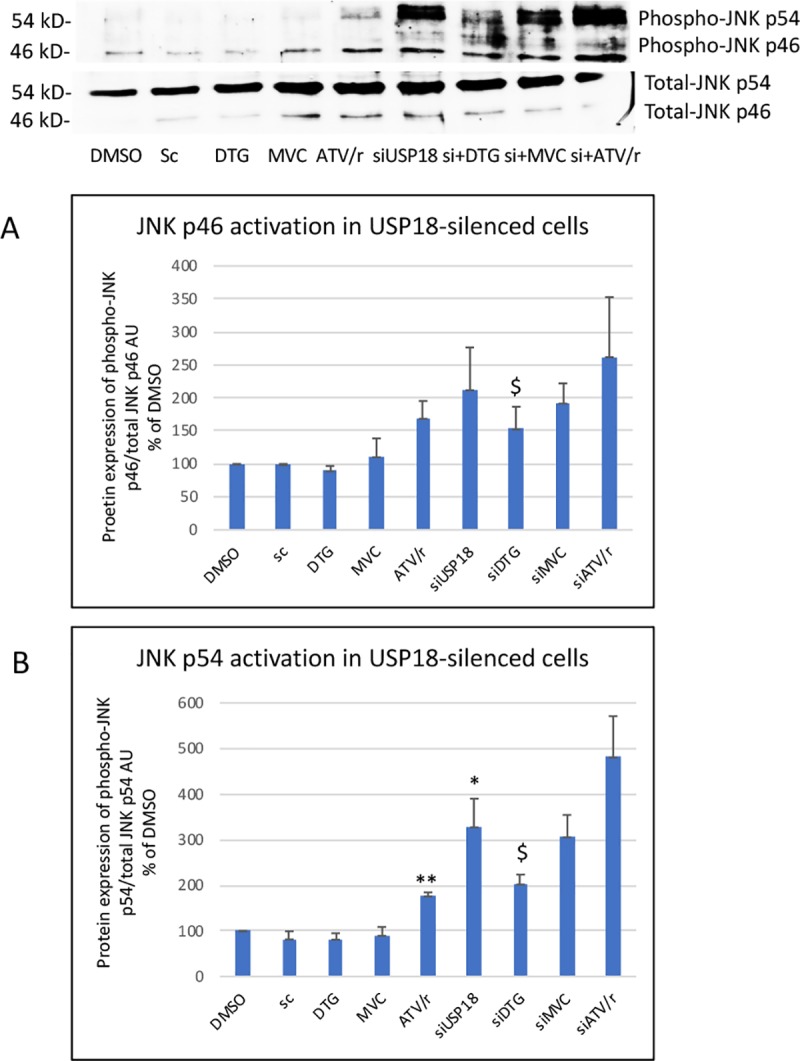
Effect of USP18 silencing on the ability of DTG, MVC and ATV/r to modulate basal activity of JNK1(p46) and 2(p54) involved in insulin resistance. A representative Western blot of phospho-JNK p46 and p54 and of total JNK p46 and p54 is shown. Both JNK activations were evaluated by the ratio of the phospho- to the total form for each enzyme. The histograms represent the mean+/-SEM value as compared to the DMSO control set at 100% of 3 independent experiments. Si-DMSO: control silenced for USP18, Si-DTG: DTG-treated cells silenced for USP18, Si-MVC: MVC-treated cells silenced for USP18, Si-ATV/r: ATV/r-treated cells silenced for USP18. The comparisons used Welch’s correction of Student T-test. ** p<0.01 versus control (DMSO) $ p< 0.05 versus Si-USP18 (Si-DMSO).

We also analyzed steps involved in insulin resistance upstream of AKT, at the level of the TGFβ-activated kinase (TAK-1) and of the two Jun kinases, JNK1(p46) and JNK2 (p54) by evaluating the ratio between the phosphorylated and the total form of each enzyme [39, 43]. USP18 silencing increased the basal level of activation of TAK-1, JNK1 and JNK2 (Figs 11B and 12). In silenced cells, in accordance with their effect on insulin sensitivity, DTG decreased TAK-1, JNK1 and JNK2 activation while ATV/r tended to increase this level (increased activation in each experiment but large inter-experiment variations in the % of increase). Therefore, DTG improved and ATV/r worsened insulin sensitivity in USP18-silenced cells indicating that their effects were not dependent on USP18. MVC did not alter insulin signaling.

## 4. Discussion

We show here that HIV antiretrovirals from three classes differently affected inflammation, senescence and insulin sensitivity in endothelial cells. The PI ATV/r enhanced inflammation, senescence and insulin resistance while the INSTI DTG had opposite effects. The CCR5 inhibitor MVC was mildly anti-inflammatory and reduced senescence with no effect on insulin sensitivity.

The ability of PIs to increase the cardiovascular risk has been previously addressed, mainly for ritonavir-boosted lopinavir and ritonavir-boosted darunavir [7, 9]. In in vitro studies, we and others have observed that the different protease inhibitors were able to enhance inflammation and induce endothelial dysfunction to different extents, with lopinavir presenting the worse profile, ATV/r exerting milder and darunavir/r even milder effects [31].

Regarding INSTI, a recent clinical study indicated that their use was associated with decreased occurrence of cardio-vascular outcomes as compared to other ART (PI or non-nucleoside analogue reverse transcriptase inhibitors) [16], even if the individual INSTI were not analyzed in this study. Also, all studies evaluating switch strategies from PI towards INSTI reported improved lipid levels (total and LDL cholesterol, triglycerides) after the switch [13] [4, 11, 12] [14, 44]. We previously reported that, in endothelial cells, raltegravir had no effect on endothelial function while DTG improved it [28].

Few clinical studies, including a low number of patients, have evaluated the effect of MVC intensification on vascular parameters. In 15 aviremic PI-treated HIV-infected patients, MVC intensification resulted in a reduction in intima-media thickness and pulse wave velocity, supporting a reduction in the cardiovascular risk and an improvement in endothelial function [27]. As well, MVC intensification in 22 HIV-infected patients at high cardiovascular risk improved several markers for cardiovascular risk, endothelial dysfunction, and arterial stiffness [26].

The ability of PIs or INSTI to modulate inflammation has been evaluated in several studies switching off PIs to INSTI. However, in that setting, it is difficult to address the respective role of each antiretroviral class. In the SPIRAL study, in which patients were switched off PIs to raltegravir, levels of CRP, monocyte chemoattractant protein 1 (MCP-1), TNF-α and IL-6 were decreased [44]. As well, patients switched off PIs (mainly ATV/r and ritonavir-boosted darunavir) to raltegravir or DTG presented markedly decreased levels of IL-6 [23]. In the NEAT022 study, switching off contemporary PIs to DTG resulted in decreased levels of soluble-CD14 with a tendency for CRP and oxidized-LDL [13]. Patients from ETRAL switched off contemporary PIs to raltegravir plus etravirine presented decreased levels of interferon-gamma inducible protein 10 (IP10) and solubleCD14 but increased levels of D-dimer [12]. Accordingly, in the present study, we observed that ATV/r exerted pro-inflammatory and DTG anti-inflammatory effects and we have previously reported that raltegravir did not modify inflammation in endothelial cells [28].

One study on MVC intensification reported that IL-6, sICAM-1 and sVCAM-1 levels were decreased [27] but the inflammatory markers remained unmodified in another one [26], suggesting that the effect of MVC on inflammation is probably limited. We have previously shown a mild anti-inflammatory effect of the drug on endothelial cells that was also found in the present work[28].

Several PIs have been associated with increased insulin resistance. In healthy non-infected controls, a 10-day treatment with ritonavir-boosted lopinavir was associated with markedly increased insulin resistance and a similar trend was reported for ATV/r to a lesser extent [45]. We report here that ATV/r worsened insulin resistance in endothelial cells.

Regarding INSTI, clinical data on insulin sensitivity are discrepant. In the SPIRAL study, switching off PIs to raltegravir improved insulin sensitivity [44]. As well, patients switched off PIs to raltegravir or DTG presented improved insulin sensitivity [23]. Otherwise, when evaluating either ART-naïve or ART-experienced patients, insulin resistance (evaluated by the HOMA-IR index) increased similarly in subjects receiving raltegravir or DTG and in those receiving other antiretroviral regimens [13, 24, 25]. In the ETRAL study, PI-controlled HIV-infected patients switched to a dual raltegravir-etravirine therapy gained fat and increased their insulin level, indicating increased insulin resistance[12].

Greater weight gain has been reported both in cohort studies and clinical trials with INSTIs, as compared to PIs and non-nucleoside analogue reverse transcriptase inhibitors, a worrisome outcome [17, 18] [4, 12, 19, 21, 22, 46, 47], }. This weight gain was more marked with DTG than raltegravir and in a recent study equivalent between DTG and bictegravir[48]. Weight gain following ART initiation could be attributable in part to a “return to health” phenomenon in patients with severe HIV infection, but this is not the case for switched patients. Weight gain is also associated with personal factors as sex, age and ethnicity [4, 49]. This increased weight has been associated, for DTG and raltegravir, with a decreased circulating level of the adipokine adiponectin [13, 50], an insulin-sensitizing and anti-inflammatory adipokine, suggesting a deleterious impact of these INSTIs on adipose tissue. This situation could suggest that INSTI differently impact different tissues. They could exert neutral or favorable effects on the vascular wall and adverse effects on adipose tissue. Moreover, the nucleoside reverse transcriptase inhibitor backbone could also be involved in fat gain as shown by the fat gain-favoring effect of tenofovir alafenamide (TAF) plus DTG as compared to tenofovir disoproxil fumarate (TDF) plus DTG[17].

We decided to further decipher the pathways involved in the effect of DTG, MVC and ATV/r in endothelial cells. At first, we evaluated the role of the SIRT-1 pathway, since we have previously observed that the SIRT-1 protein level was modified by the different antiretrovirals, with an effect opposite to their ability to activate NFκB [28]. Importantly, SIRT-1 has been shown to play an important role in endothelial cells, being able to reduce the production of reactive oxygen species, prevent premature senescence and inhibit NFκB [51–53]. Therefore, we thought that SIRT-1 was a credible candidate to explain modified NFκB activity in response to antiretroviral molecules. SIRT-1 activation decreased the level of inflammation, as expected, and the reverse was observed when SIRT-1 was inhibited, confirming the role of SIRT-1 in the basal inflammatory state in endothelial cells. However, the ability of DTG to decrease basal inflammation in cells exposed to the SIRT-1 inhibitor was preserved, indicating that DTG and SIRT-1 were using different pathways to decrease inflammation. In this setting, MVC had no effect on inflammation.

To better approach the mechanisms involved in the drug effect, we performed a non-targeted transcriptomic analysis and evaluated the level of the genes altered by the different drugs. Among different genes of interest, we selected, at first, USP18, a specific ISG15 isopeptidase and an inhibitor of the interferon type 1 signal [54], which exerts different protease-dependent and independent effects. The ability of USP18 to decrease inflammation has been previously reported in T cell [40, 41]. USP18 was shown to inhibit the NFκB pathway and TAK1. However, the catalytic deubiquitinating activity of USP18 was required for TAK-1 but not for NFκB inhibition, indicating that different signaling pathways were involved. Accordingly, since TAK1 is required for JNK activation, leading to insulin resistance, and since USP18 inhibits TAK-1, an insulin-sensitizing effect of USP18 has been shown in the liver [39, 43].

By using the siRNA strategy, we silenced the gene and reduced by 60–70% the level of the USP18 protein. Interestingly, the level of inflammation and insulin resistance was increased in cells with reduced USP18 level. Therefore, our results, which demonstrate the ability of USP18 to inhibit TAK-1 and JNK activation resulting in enhanced insulin sensitivity, agree with other studies obtained in T lymphocytes and the liver [39–41]. Interestingly, we observe that the effect of DTG and ATV/r on inflammation required USP18 but that USP18 was dispensable for their effect on TAK1, JNK and AKT activation, indicating that they affected insulin signaling independently of USP18.

We have previously shown that some ART were able to affect senescence in endothelial cells[28], DTG and MVC preventing and ATV/r increasing the senescence level. We show here, in addition, that USP18 was also able to prevent senescence, since its basal level was increased in USP18-silenced cells. Moreover, in these cells, MVC, but not DTG and ATV/r, was still able to reduce the level of senescence markers. It will be interesting, in a future work, to analyze the interactions between inflammation and senescence in this setting.

**Fig 13:**
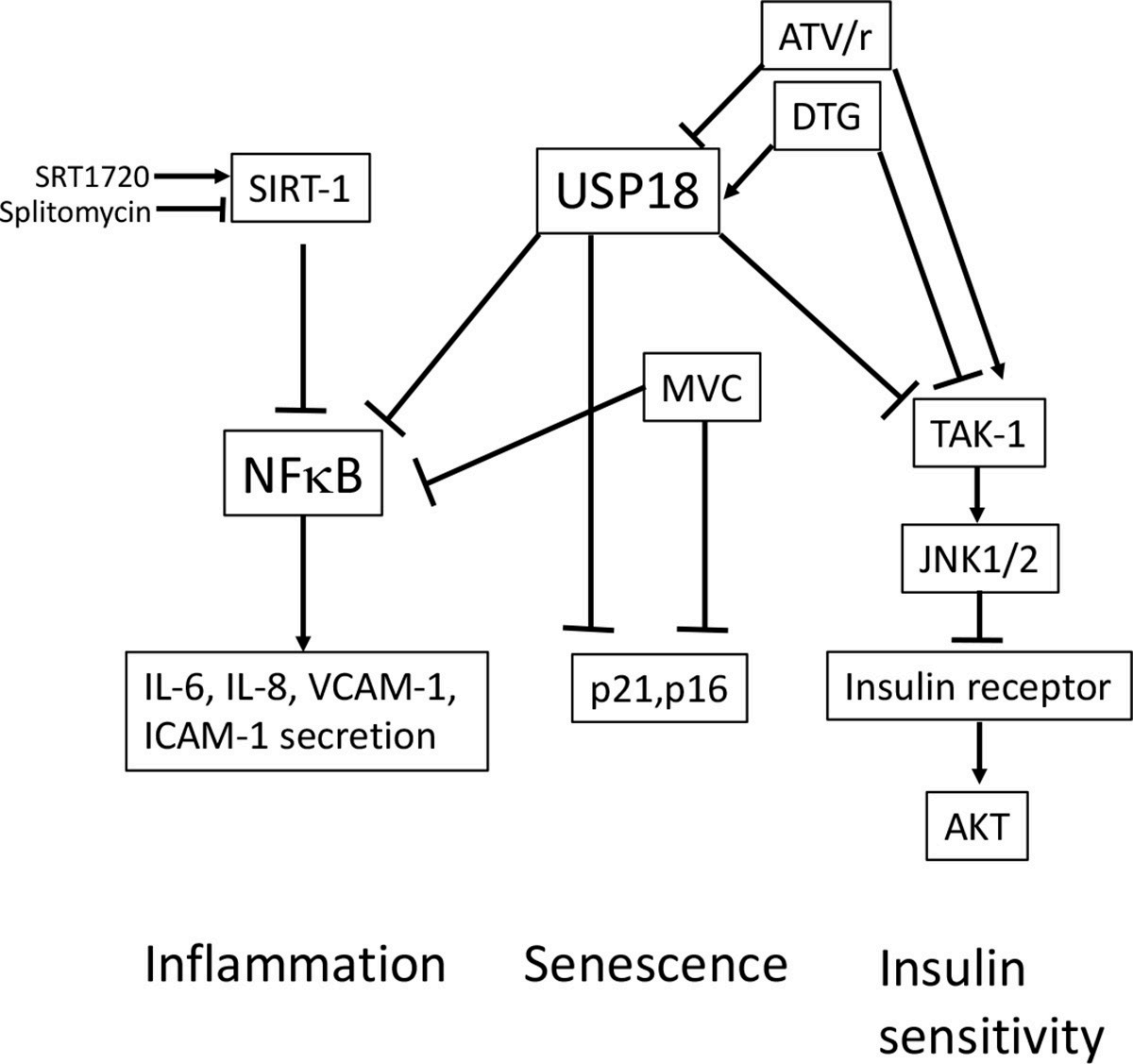
Hypothetical view of the signaling pathways used by DTG, MVC and ATV/r to alter inflammation, senescence and insulin sensitivity in coronary artery endothelial cells. We propose that USP18 is differently involved in inflammatory, senescence and insulin-sensitizing pathways. The effects of DTG and ATV/r on inflammation and senescence could involve USP18 but not their effects on insulin sensitivity. MVC signals on inflammation and senescence independently of USP18 and does not modify insulin sensitivity. The possible relationships between inflammation and senescence need to be further evaluated.

Therefore, DTG and ATV/r exerted opposite effects on the same three pathways, insulin resistance, senescence and inflammation, respectively independent and dependent on USP18. The effect of MVC on inflammation and senescence was independent on USP18. A schematic view of these different pathways is presented in Fig 13.

Our work has limitations. We used an in vitro model of endothelial cells and our results need to be validated in the clinical situation. Nevertheless, in vitro models allow to analyze separately different tissues and drugs, while, in the clinical situation, the evaluation is more global. Therefore, to perform in vitro studies could be relevant in the situation of INSTI, since INSTI probably present discrepant adverse effects, with a possible deleterious impact on adipose tissue but a neutral or favorable one on lipids and vascular wall. Indeed, we previously observed that raltegravir was neutral and we confirm here that DTG exerted favorable effects in endothelial cells. We provide here results suggesting links between different signaling pathway and enlightening the importance of USP18 in several of these pathways. Further studies will be important to better decipher these interactions, in particular the link between SIRT-1, USP18, inflammation and senescence.

## 5. Conclusions

We have evaluated the effect of individual drugs from three ART classes on endothelial cells as a surrogate of arterial wall. MVC exerted no or mild effects on insulin sensitivity, senescence and inflammation. DTG presented a favorable profile regarding these three parameters conversely to ATV/r. The effects of the drugs signaled through different pathways, and in particular, involved USP18 for the inflammatory and senescence pathways. Therefore, this enzyme could represent a potential target to modulate inflammation and senescence in ART-controlled patients.

## Supporting information

S1 Fig Effect of the antiretrovirals on the cellular protein level of CCR5. A representative blot of CCR5 and tubulin as a loading control is shown. The histograms represent the mean+/-SEM value as compared to the DMSO control set at 100% of 3 independent experiments. Sc scramble control.
(TIF)

S2 Fig Raw data of Western blots 1. (PDF)

S3 Fig Raw data of Western blots 2. (PDF)

## References

[1] Duffau P, Wittkop L, Lazaro E, le Marec F, Cognet C, Blanco P, et al Association of immune-activation and senescence markers with non-AIDS-defining comorbidities in HIV-suppressed patients. AIDS. 2015;29(16):2099–108. 10.1097/QAD.0000000000000807 . 26544576

[2] Bastard JP, Couffignal C, Fellahi S, Bard JM, Mentre F, Salmon D, et al Diabetes and dyslipidaemia are associated with oxidative stress independently of inflammation in long-term antiretroviral-treated HIV-infected patients. Diabetes Metab. 2019 Epub 2019/03/14. 10.1016/j.diabet.2019.02.008 . 30862472

[3] Schouten J, Wit FW, Stolte IG, Kootstra NA, van der Valk M, Geerlings SE, et al Cross-sectional comparison of the prevalence of age-associated comorbidities and their risk factors between HIV-infected and uninfected individuals: the AGEhIV cohort study. Clinical infectious diseases: an official publication of the Infectious Diseases Society of America. 2014;59(12):1787–97. 10.1093/cid/ciu701 . 25182245

[4] Lagathu C, Bereziat V, Gorwood J, Fellahi S, Bastard JP, Vigouroux C, et al Metabolic complications affecting adipose tissue, lipid and glucose metabolism associated with HIV antiretroviral treatment. Expert Opin Drug Saf. 2019;18(9):829–40. Epub 2019/07/16. 10.1080/14740338.2019.1644317 . 31304808

[5] Group DADS, Friis-Moller N, Reiss P, Sabin CA, Weber R, Monforte A, et al Class of antiretroviral drugs and the risk of myocardial infarction. N Engl J Med. 2007;356(17):1723–35. 10.1056/NEJMoa062744 . 17460226

[6] Eckard AR, Meissner EG, Singh I, McComsey GA. Cardiovascular Disease, Statins, and HIV. The Journal of infectious diseases. 2016;214 Suppl 2:S83–92. 10.1093/infdis/jiw288 27625435 PMC5021243

[7] Boccara F, Lang S, Meuleman C, Ederhy S, Mary-Krause M, Costagliola D, et al HIV and coronary heart disease: time for a better understanding. J Am Coll Cardiol. 2013;61(5):511–23. 10.1016/j.jacc.2012.06.063 . 23369416

[8] Lang S, Mary-Krause M, Cotte L, Gilquin J, Partisani M, Simon A, et al Impact of individual antiretroviral drugs on the risk of myocardial infarction in human immunodeficiency virus-infected patients: a case-control study nested within the French Hospital Database on HIV ANRS cohort CO4. Arch Intern Med. 2010;170(14):1228–38. 10.1001/archinternmed.2010.197 . 20660842

[9] Ryom L, Lundgren JD, El-Sadr W, Reiss P, Kirk O, Law M, et al Cardiovascular disease and use of contemporary protease inhibitors: the D:A:D international prospective multicohort study. Lancet HIV. 2018;5(6):e291–e300. Epub 2018/05/08. 10.1016/S2352-3018(18)30043-2 . 29731407

[10] Gupta N, Singh T, Chaudhary R, Garg SK, Sandhu GS, Mittal V, et al Bilirubin in coronary artery disease: Cytotoxic or protective? World J Gastrointest Pharmacol Ther. 2016;7(4):469–76. 10.4292/wjgpt.v7.i4.469 27867680 PMC5095566

[11] Brenner BG, Baril JG. Limiting cardiovascular events associated with HIV and antiretroviral therapy. AIDS. 2017;31(18):2551–3. Epub 2017/11/10. 10.1097/QAD.0000000000001676 . 29120901

[12] Katlama C, Assoumou L, Valantin MA, Soulie C, Martinez E, Beniguel L, et al Dual therapy combining raltegravir with etravirine maintains a high level of viral suppression over 96 weeks in long-term experienced HIV-infected individuals over 45 years on a PI-based regimen: results from the Phase II ANRS 163 ETRAL study. The Journal of antimicrobial chemotherapy. 2019;74(9):2742–51. Epub 2019/07/04. 10.1093/jac/dkz224 . 31269208

[13] Martinez E, Assoumou L, Moyle G, Waters L, Johnson M, Domingo P, et al 48-week changes in biomarkers in subjects with high cardiovascular risk boosted switching from ritonavir-protease inhibitors to dolutegravir: the NEAT022 study. Journal of the International AIDS Society. 2018;21(S8):e25187.30362663

[14] Gatell JM, Assoumou L, Moyle G, Waters L, Johnson M, Domingo P, et al Immediate Versus Deferred Switching From a Boosted Protease Inhibitor-based Regimen to a Dolutegravir-based Regimen in Virologically Suppressed Patients With High Cardiovascular Risk or Age >/ = 50 Years: Final 96-Week Results of the NEAT022 Study. Clinical infectious diseases: an official publication of the Infectious Diseases Society of America. 2019;68(4):597–606. Epub 2018/06/19. 10.1093/cid/ciy505 . 29912307

[15] Hill AM, Mitchell N, Hughes S, Pozniak AL. Risks of cardiovascular or central nervous system adverse events and immune reconstitution inflammatory syndrome, for dolutegravir versus other antiretrovirals: meta-analysis of randomized trials. Curr Opin HIV AIDS. 2018;13(2):102–11. Epub 2017/12/27. 10.1097/COH.0000000000000445 . 29278532

[16] O'Halloran JA, Sahrmann J, Butler AM, Olsen MA, Powderly WG, editors. LOWER CARDIOVASCULAR DISEASE RISK ASSOCIATED WITH INTEGRASE INHIBITORS. CROI; 2019; Seattle.

[17] Venter WDF, Moorhouse M, Sokhela S, Fairlie L, Mashabane N, Masenya M, et al Dolutegravir plus Two Different Prodrugs of Tenofovir to Treat HIV. N Engl J Med. 2019;381(9):803–15. Epub 2019/07/25. 10.1056/NEJMoa1902824 . 31339677

[18] Hill A, Waters L, Pozniak A. Are new antiretroviral treatments increasing the risks of clinical obesity? J Virus Erad. 2019;5(1):41–3. Epub 2019/02/26. 30800425 10.1016/S2055-6640(20)30277-6 PMC6362910

[19] Bourgi K, Jenkins CA, Rebeiro PF, Lake JE, Moore, Mathews WC, et al, editors. GREATER WEIGHT GAIN AMONG TREATMENT-NAIVE PERSONS STARTING INTEGRASE INHIBITORS. CROI; 2019; Seattle.

[20] Lake JE, Wu K, Erlandson KM, Bares SH, Debroy P, Godfrey C, et al, editors. RISK FACTORS FOR EXCESS WEIGHT GAIN FOLLOWING SWITCH TO INTEGRASE INHIBITOR–BASED ART. CROI; 2019; Seattle. 10.1093/cid/ciaa177 PMC7713693 32099991

[21] Bourgi K, Rebeiro PF, Turner M, Castilho JL, Hulgan T, Raffanti SP, et al Greater Weight Gain in Treatment Naive Persons Starting Dolutegravir-Based Antiretroviral Therapy. Clinical infectious diseases: an official publication of the Infectious Diseases Society of America. 2019 Epub 2019/05/18. 10.1093/cid/ciz407 . 31100116 PMC8205610

[22] Bernardino JI, Mocroft A, Wallet C, de Wit S, Katlama C, Reiss P, et al Body composition and adipokines changes after initial treatment with darunavir-ritonavir plus either raltegravir or tenofovir disoproxil fumarate-emtricitabine: A substudy of the NEAT001/ANRS143 randomised trial. PloS one. 2019;14(1):e0209911 Epub 2019/01/29. 10.1371/journal.pone.0209911 ; PubMed Central PMCID: PMC6349314 30689664 PMC6349314

[23] Calza L, Colangeli V, Borderi M, Coladonato S, Tazza B, Bon I, et al Improvement in insulin sensitivity and serum leptin concentration after the switch from a ritonavir-boosted PI to raltegravir or dolutegravir in non-diabetic HIV-infected patients. The Journal of antimicrobial chemotherapy. 2019;74(3):731–8. Epub 2018/12/13. 10.1093/jac/dky507 . 30541118

[24] Dirajlal-Fargo S, Moser C, Brown TT, Kelesidis T, Dube MP, Stein JH, et al Changes in Insulin Resistance After Initiation of Raltegravir or Protease Inhibitors With Tenofovir-Emtricitabine: AIDS Clinical Trials Group A5260s. Open Forum Infect Dis. 2016;3(3):ofw174 Epub 2016/10/06. 10.1093/ofid/ofw174 27704026 PMC5047417

[25] Lo J, Oyee J, Crawford M, Grove R, DeMasi R, Curtis L, et al, editors. DOLUTEGRAVIR AND INSULIN RESISTANCE. CROI; 2019; Seattle.

[26] Francisci D, Pirro M, Schiaroli E, Mannarino MR, Cipriani S, Bianconi V, et al Maraviroc Intensification Modulates Atherosclerotic Progression in HIV-Suppressed Patients at High Cardiovascular Risk. A Randomized, Crossover Pilot Study. Open Forum Infect Dis. 2019;6(4):ofz112 Epub 2019/04/11. 10.1093/ofid/ofz112 30968058 PMC6446135

[27] Piconi S, Pocaterra D, Rainone V, Cossu M, Masetti M, Rizzardini G, et al Maraviroc Reduces Arterial Stiffness in PI-Treated HIV-infected Patients. Scientific reports. 2016;6:28853 10.1038/srep28853 27352838 PMC4926207

[28] Afonso P, Auclair M, Caron-Debarle M, Capeau J. Impact of CCR5, integrase and protease inhibitors on human endothelial cell function, stress, inflammation and senescence. Antiviral therapy. 2017;22(8):645–57. Epub 2017/03/30. 10.3851/IMP3160 . 28350300

[29] Berger O, Gan X, Gujuluva C, Burns AR, Sulur G, Stins M, et al CXC and CC chemokine receptors on coronary and brain endothelia. Mol Med. 1999;5(12):795–805. Epub 2000/02/10. 10666479 PMC2230493

[30] Maguire JJ, Jones KL, Kuc RE, Clarke MC, Bennett MR, Davenport AP. The CCR5 chemokine receptor mediates vasoconstriction and stimulates intimal hyperplasia in human vessels in vitro. Cardiovascular research. 2014;101(3):513–21. 10.1093/cvr/cvt333 24323316 PMC3928001

[31] Auclair M, Afonso P, Capel E, Caron-Debarle M, Capeau J. Impact of darunavir, atazanavir and lopinavir boosted with ritonavir on cultured human endothelial cells: beneficial effect of pravastatin. Antiviral therapy. 2014;19(8):773–82. Epub 2014/02/19. 10.3851/IMP2752 . 24535489

[32] Gano LB, Donato AJ, Pasha HM, Hearon CM Jr., Sindler AL, Seals DR. The SIRT1 activator SRT1720 reverses vascular endothelial dysfunction, excessive superoxide production, and inflammation with aging in mice. Am J Physiol Heart Circ Physiol. 2014;307(12):H1754–63. Epub 2014/10/19. 10.1152/ajpheart.00377.2014 25326534 PMC4269699

[33] Li RL, Lu ZY, Huang JJ, Qi J, Hu A, Su ZX, et al SRT1720, a SIRT1 specific activator, protected H2O2-induced senescent endothelium. Am J Transl Res. 2016;8(7):2876–88. Epub 2016/08/11. 27508009 PMC4969425

[34] Stavrou EX, Fang C, Merkulova A, Alhalabi O, Grobe N, Antoniak S, et al Reduced thrombosis in Klkb1-/- mice is mediated by increased Mas receptor, prostacyclin, Sirt1, and KLF4 and decreased tissue factor. Blood. 2015;125(4):710–9. Epub 2014/10/24. 10.1182/blood-2014-01-550285 25339356 PMC4304115

[35] Liu FC, Day YJ, Liou JT, Yu HP, Liao HR. Splitomicin inhibits fMLP-induced superoxide anion production in human neutrophils by activate cAMP/PKA signaling inhibition of ERK pathway. Eur J Pharmacol. 2012;688(1–3):68–75. Epub 2012/05/29. 10.1016/j.ejphar.2012.05.006 . 22634165

[36] Mukohda M, Stump M, Ketsawatsomkron P, Hu C, Quelle FW, Sigmund CD. Endothelial PPAR-gamma provides vascular protection from IL-1beta-induced oxidative stress. Am J Physiol Heart Circ Physiol. 2016;310(1):H39–48. Epub 2015/11/15. 10.1152/ajpheart.00490.2015 26566726 PMC4796462

[37] de Kerdanet M, Caron-Debarle M, Nivot S, Gaillot T, Lascols O, Fremont B, et al Ten-year improvement of insulin resistance and growth with recombinant human insulin-like growth factor 1 in a patient with insulin receptor mutations resulting in leprechaunism. Diabetes Metab. 2015;41(4):331–7. Epub 2014/12/04. 10.1016/j.diabet.2014.11.001 . 25465274

[38] Auclair M, Vigouroux C, Desbois-Mouthon C, Deibener J, Kaminski P, Lascols O, et al Antiinsulin receptor autoantibodies induce insulin receptors to constitutively associate with insulin receptor substrate-1 and -2 and cause severe cell resistance to both insulin and insulin-like growth factor I. The Journal of clinical endocrinology and metabolism. 1999;84(9):3197–206. Epub 1999/09/16. 10.1210/jcem.84.9.5965 . 10487687

[39] An S, Zhao LP, Shen LJ, Wang S, Zhang K, Qi Y, et al USP18 protects against hepatic steatosis and insulin resistance through its deubiquitinating activity. Hepatology. 2017;66(6):1866–84. Epub 2017/07/19. 10.1002/hep.29375 . 28718215

[40] Liu X, Li H, Zhong B, Blonska M, Gorjestani S, Yan M, et al USP18 inhibits NF-kappaB and NFAT activation during Th17 differentiation by deubiquitinating the TAK1-TAB1 complex. J Exp Med. 2013;210(8):1575–90. Epub 2013/07/05. 10.1084/jem.20122327 23825189 PMC3727316

[41] Yang Z, Xian H, Hu J, Tian S, Qin Y, Wang RF, et al USP18 negatively regulates NF-kappaB signaling by targeting TAK1 and NEMO for deubiquitination through distinct mechanisms. Scientific reports. 2015;5:12738 Epub 2015/08/05. 10.1038/srep12738 26240016 PMC4523862

[42] Artunc F, Schleicher E, Weigert C, Fritsche A, Stefan N, Haring HU. The impact of insulin resistance on the kidney and vasculature. Nat Rev Nephrol. 2016;12(12):721–37. Epub 2016/11/01. 10.1038/nrneph.2016.145 . 27748389

[43] Zhang Y, Wan J, Xu Z, Hua T, Sun Q. Exercise ameliorates insulin resistance via regulating TGFbeta-activated kinase 1 (TAK1)-mediated insulin signaling in liver of high-fat diet-induced obese rats. J Cell Physiol. 2019;234(5):7467–74. Epub 2018/10/28. 10.1002/jcp.27508 . 30367484

[44] Martinez E, D'Albuquerque PM, Llibre JM, Gutierrez F, Podzamczer D, Antela A, et al Changes in cardiovascular biomarkers in HIV-infected patients switching from ritonavir-boosted protease inhibitors to raltegravir. AIDS. 2012;26(18):2315–26. 10.1097/QAD.0b013e328359f29c . 23018438

[45] Noor MA, Flint OP, Maa JF, Parker RA. Effects of atazanavir/ritonavir and lopinavir/ritonavir on glucose uptake and insulin sensitivity: demonstrable differences in vitro and clinically. AIDS. 2006;20(14):1813–21. Epub 2006/09/07. 10.1097/01.aids.0000244200.11006.55 . 16954722

[46] Waters L, Assoumou L, Rusconi S, Domingo P, Gompels M, de Wit S, et al, editors. Switch to dolutegravir (DTG) from a boosted protease inhibitor (PI/r) associated with significant weight gain over 48 weeks in NEAT-022, a randomised 96-week trila. HIV Drug Therapy 2018 10 2018; Glasgow.

[47] McComsey GA, Moser C, Currier J, Ribaudo HJ, Paczuski P, Dube MP, et al Body Composition Changes After Initiation of Raltegravir or Protease Inhibitors: ACTG A5260s. Clinical infectious diseases: an official publication of the Infectious Diseases Society of America. 2016;62(7):853–62. 10.1093/cid/ciw017 26797215 PMC4787610

[48] Stellbrink HJ, Arribas JR, Stephens JL, Albrecht H, Sax PE, Maggiolo F, et al Co-formulated bictegravir, emtricitabine, and tenofovir alafenamide versus dolutegravir with emtricitabine and tenofovir alafenamide for initial treatment of HIV-1 infection: week 96 results from a randomised, double-blind, multicentre, phase 3, non-inferiority trial. Lancet HIV. 2019;6(6):e364–e72. Epub 2019/05/10. 10.1016/S2352-3018(19)30080-3 . 31068272

[49] Lake JE. The Fat of the Matter: Obesity and Visceral Adiposity in Treated HIV Infection. Current HIV/AIDS reports. 2017;14(6):211–9. Epub 2017/10/19. 10.1007/s11904-017-0368-6 29043609 PMC5694708

[50] Offor O, Utay N, Reynoso D, Somasunderam A, Currier J, Lake J. Adiponectin and the steatosis marker Chi3L1 decrease following switch to raltegravir compared to continued PI/NNRTI-based antiretroviral therapy. PloS one. 2018;13(5):e0196395 Epub 2018/05/11. 10.1371/journal.pone.0196395 29746485 PMC5944924

[51] Donato AJ, Morgan RG, Walker AE, Lesniewski LA. Cellular and molecular biology of aging endothelial cells. J Mol Cell Cardiol. 2015;89(Pt B):122–35. 10.1016/j.yjmcc.2015.01.021 25655936 PMC4522407

[52] Oellerich MF, Potente M. FOXOs and sirtuins in vascular growth, maintenance, and aging. Circ Res. 2012;110(9):1238–51. Epub 2012/04/28. 10.1161/CIRCRESAHA.111.246488 . 22539757

[53] Cencioni C, Spallotta F, Mai A, Martelli F, Farsetti A, Zeiher AM, et al Sirtuin function in aging heart and vessels. J Mol Cell Cardiol. 2015;83:55–61. Epub 2015/01/13. 10.1016/j.yjmcc.2014.12.023 . 25579854

[54] Basters A, Knobeloch KP, Fritz G. USP18—a multifunctional component in the interferon response. Biosci Rep. 2018;38(6). Epub 2018/08/22. 10.1042/BSR20180250 30126853 PMC6240716

