# Supplementary figures and images for: HIV antiretroviral drugs, dolutegravir, maraviroc and ritonavir-boosted atazanavir use different pathways to affect inflammation, senescence and insulin sensitivity in human coronary endothelial cells
Source: PLoS One. 2020 Jan 23;15(1):e0226924. doi: 10.1371/journal.pone.0226924 (PMC6977740; doi:10.1371/journal.pone.0226924)

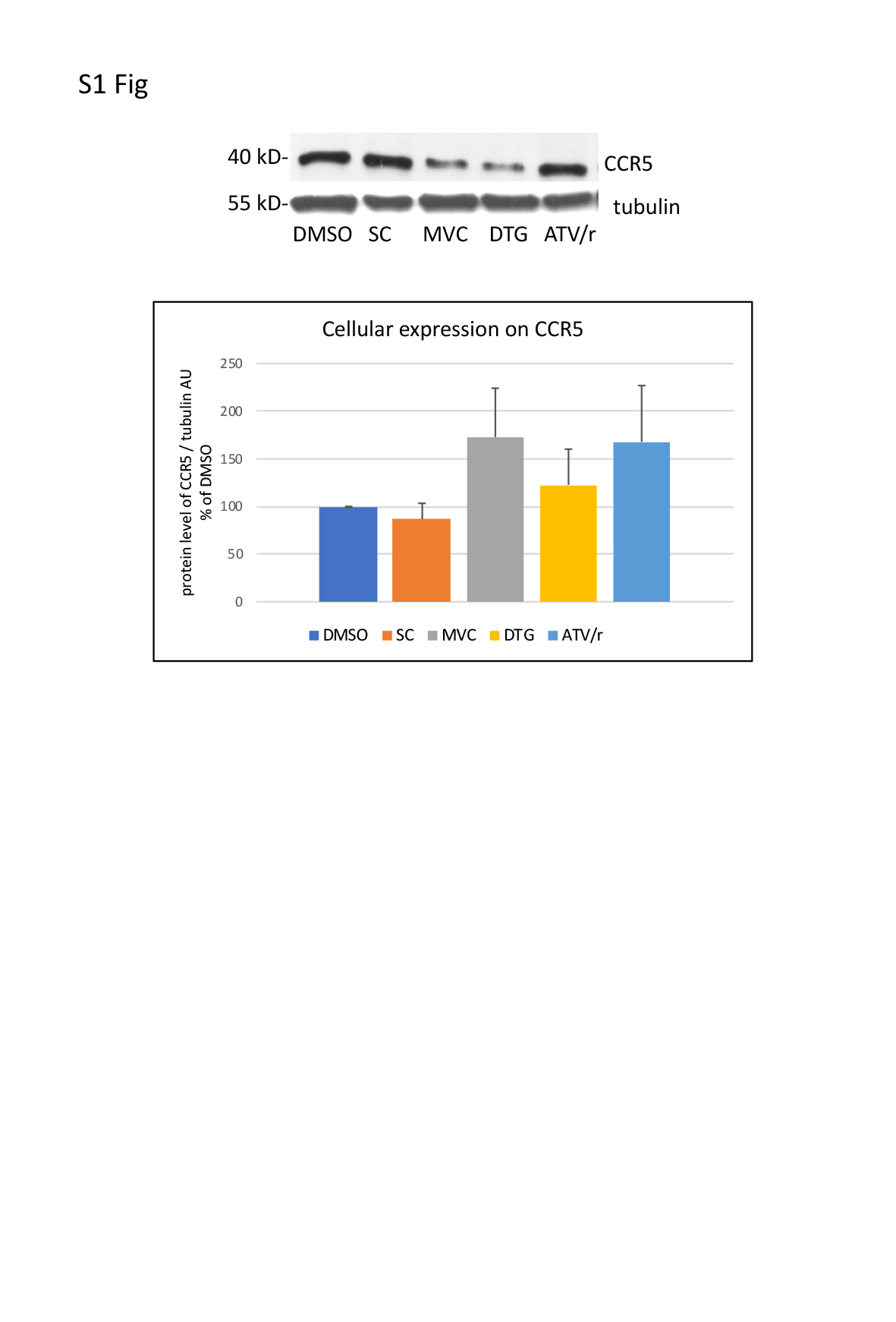

Supplement: S1 Fig — A representative blot of CCR5 and tubulin as a loading control is shown. The histograms represent the mean+/-SEM value as compared to the DMSO control set at 100% of 3 independent experiments. Sc scramble control. (TIF) [file pone.0226924.s001.tif]

Figure 1

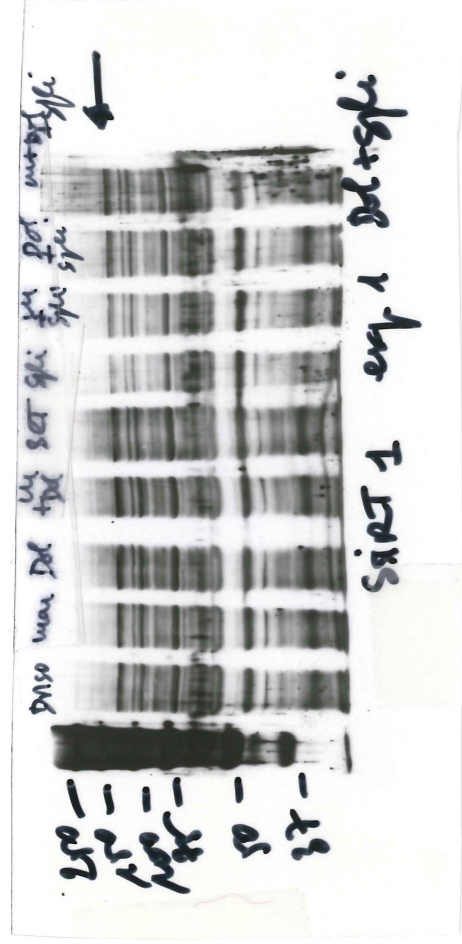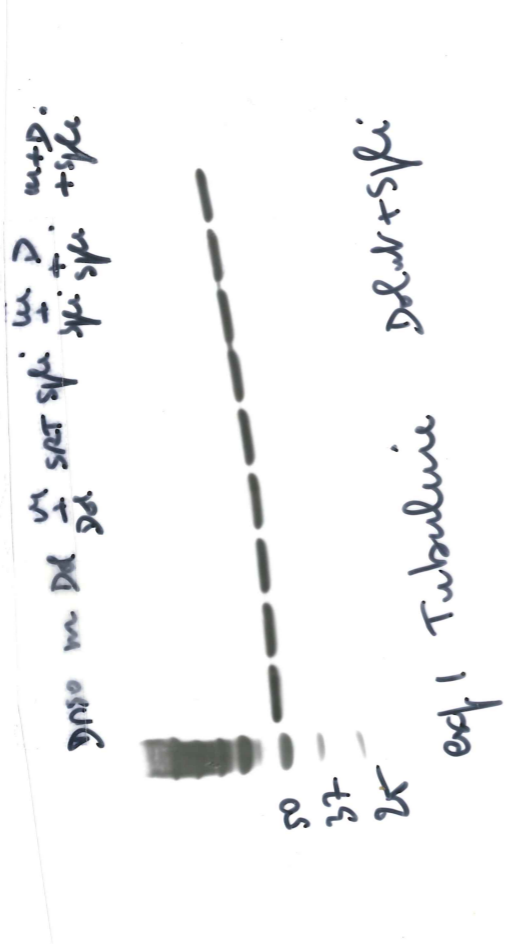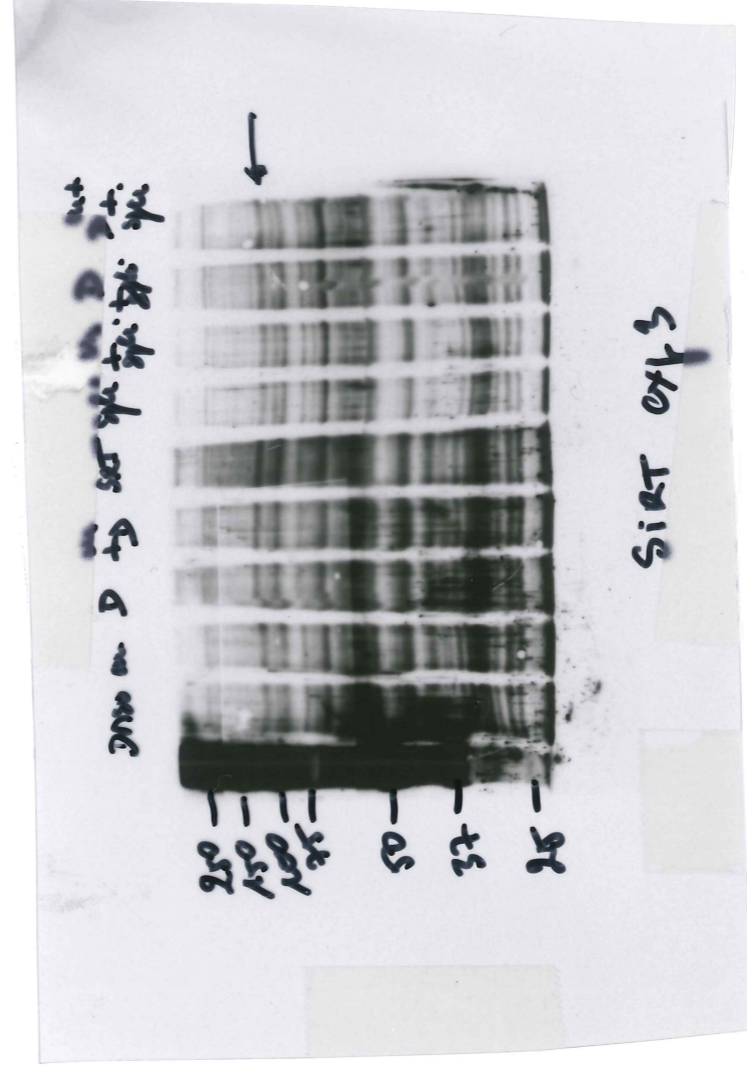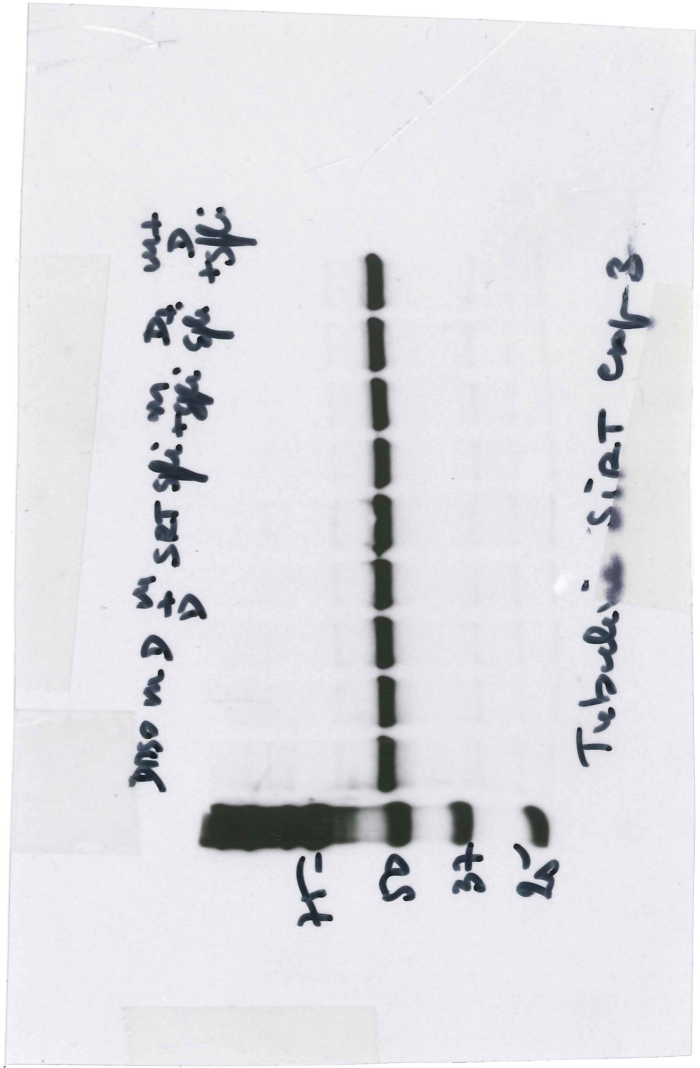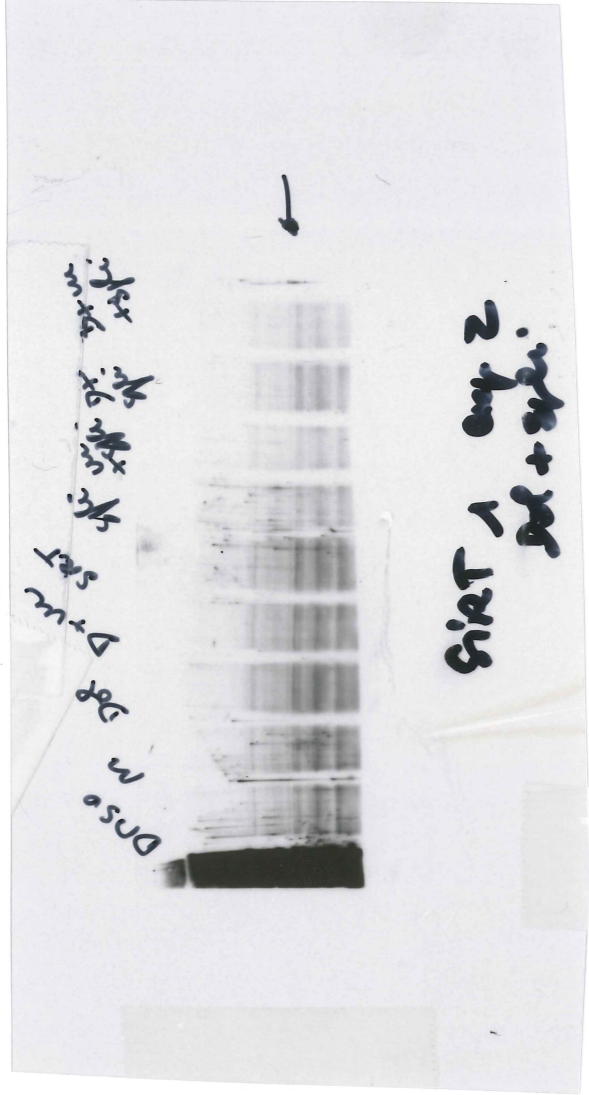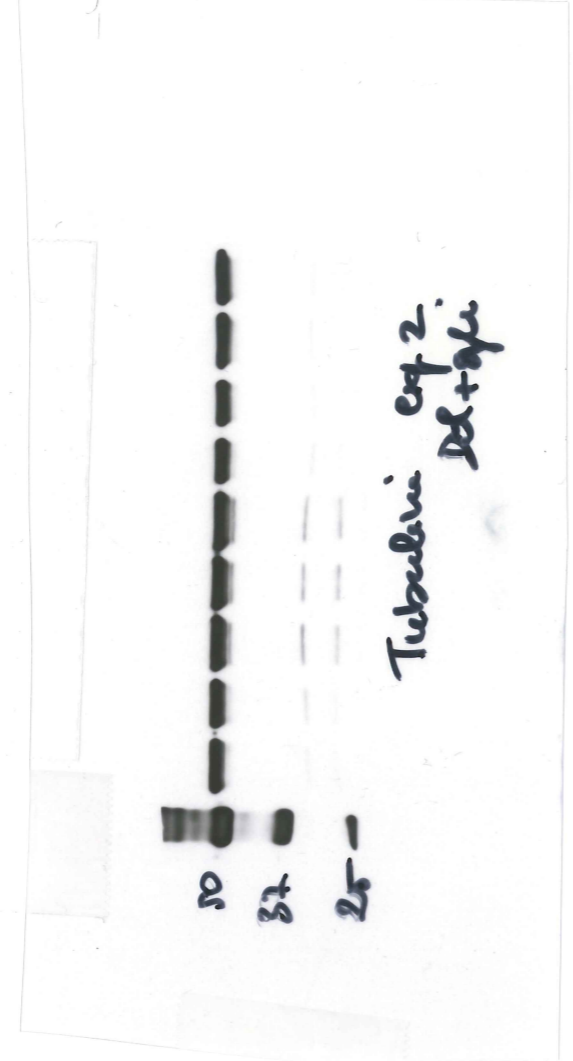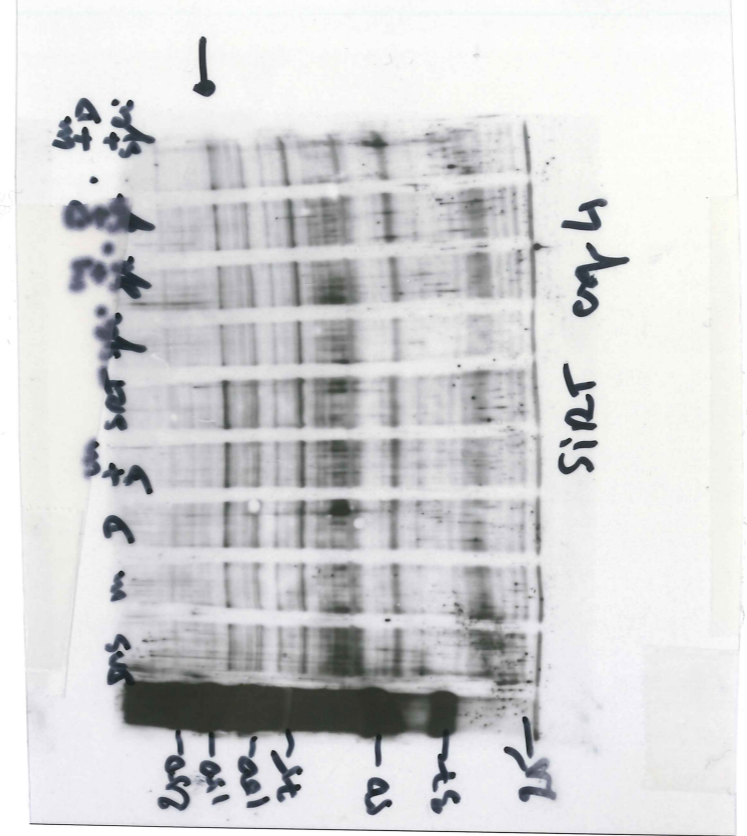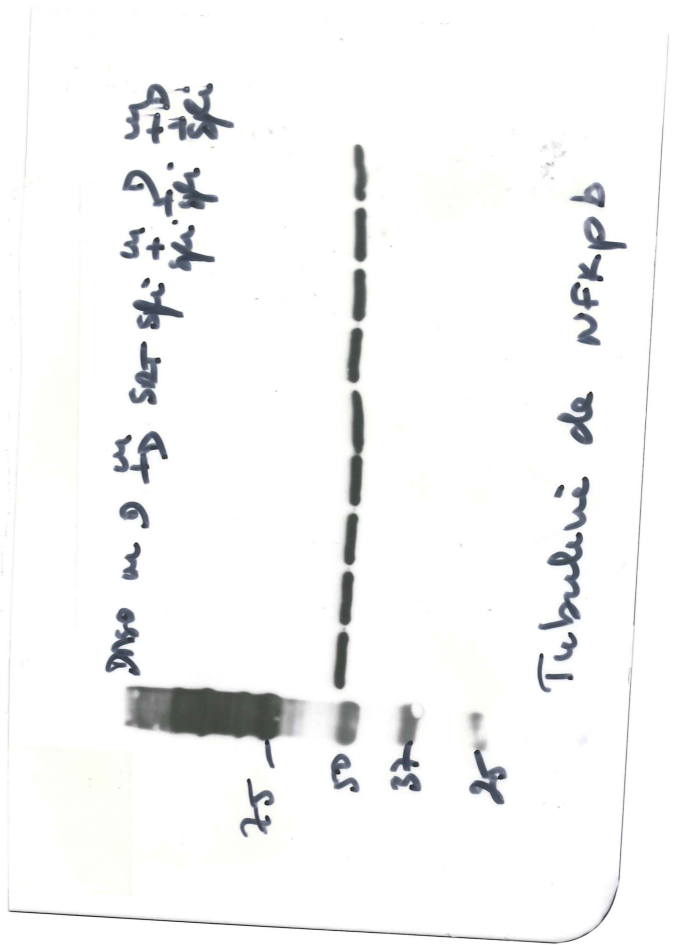



Figure 5A

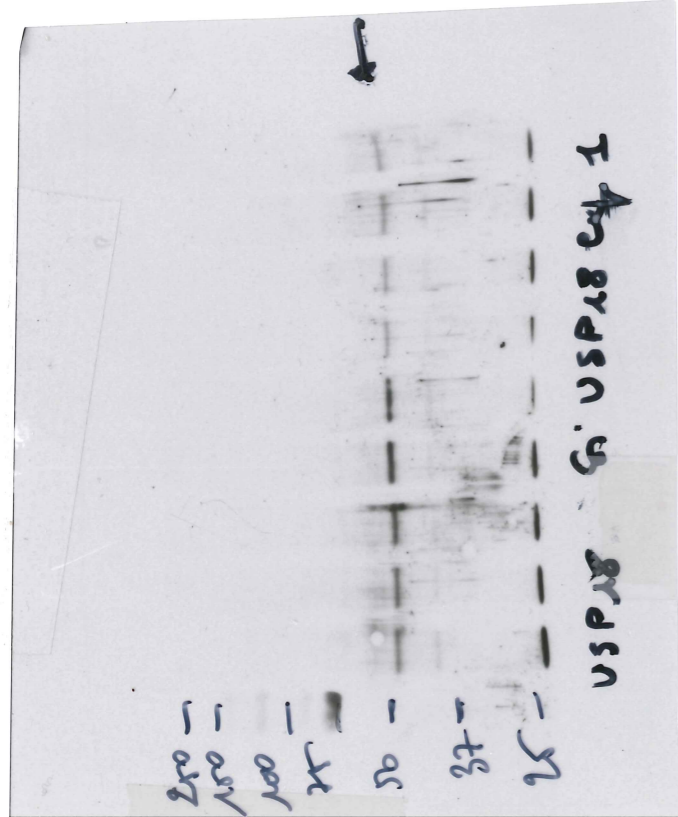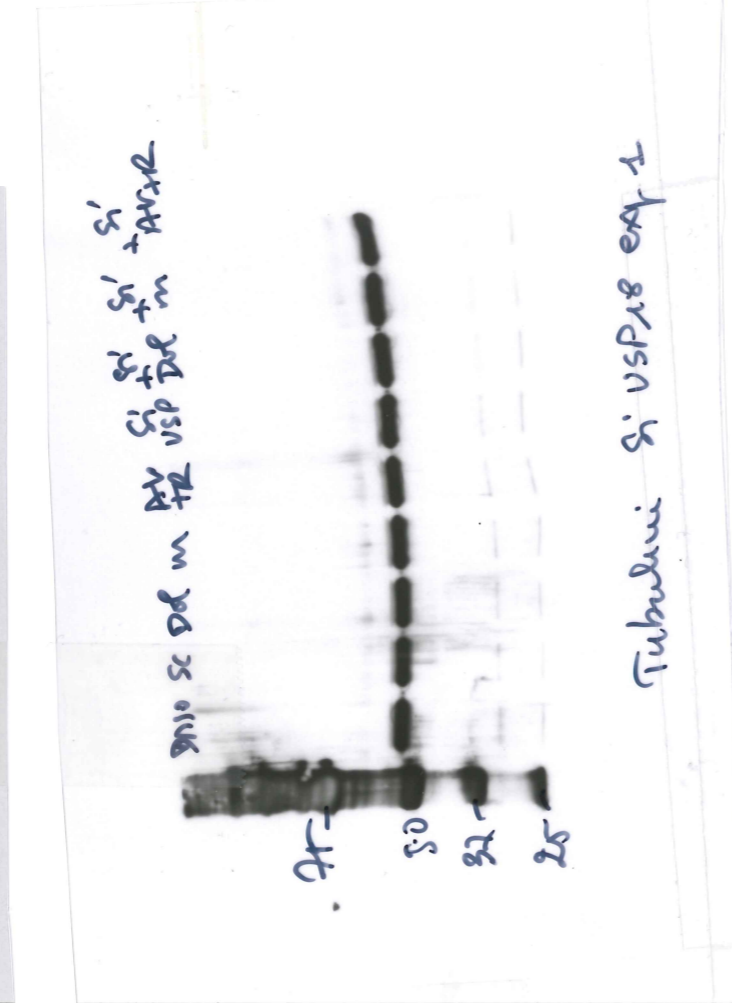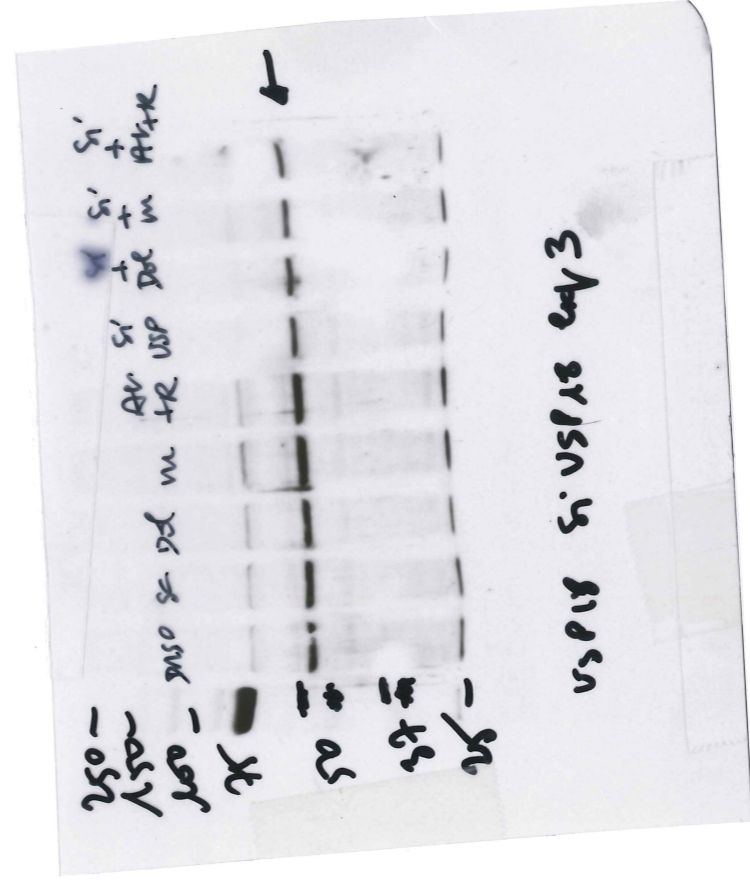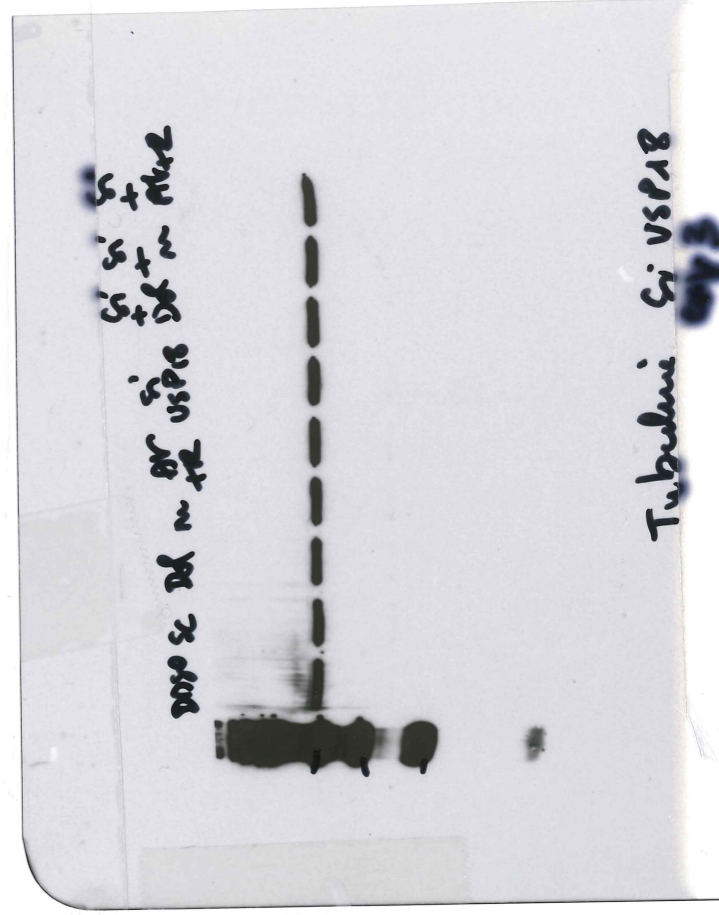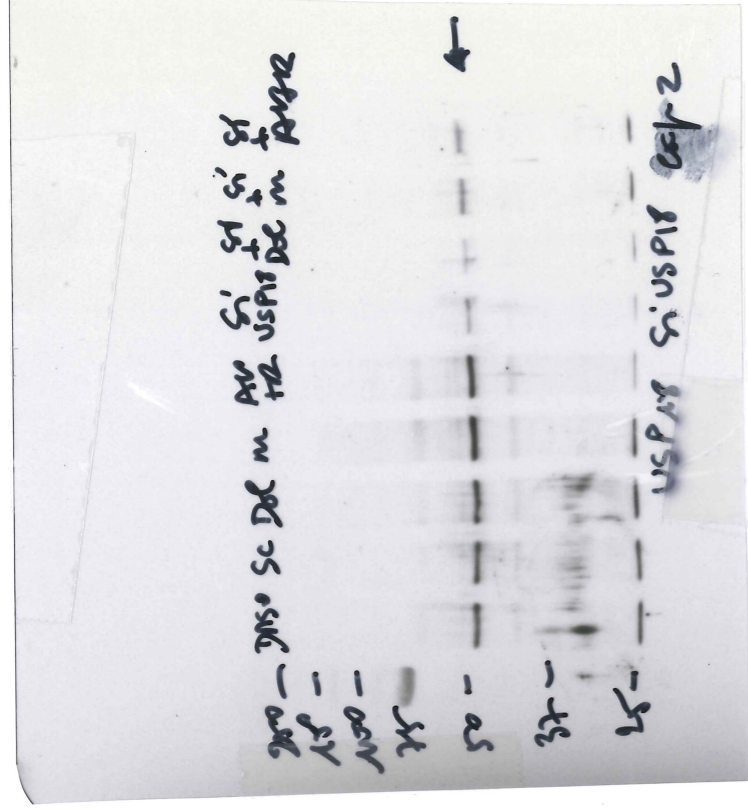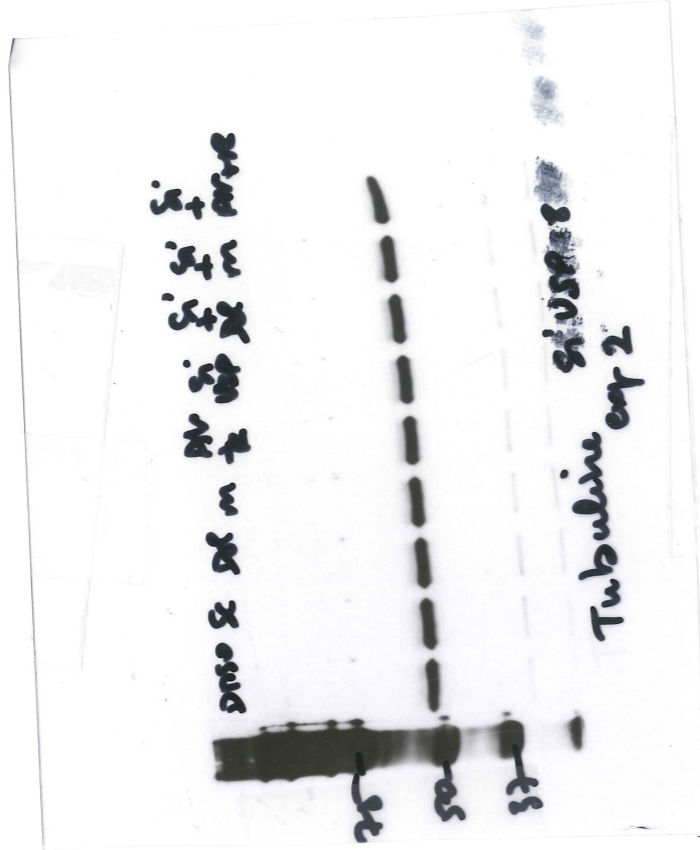

Figure 5B

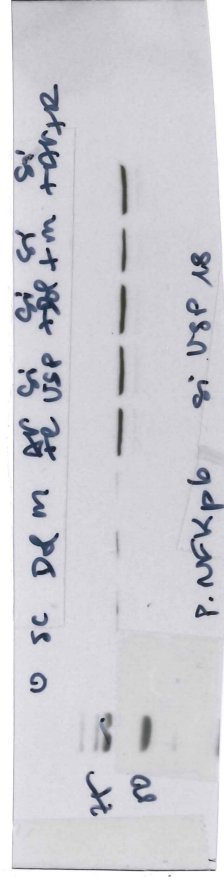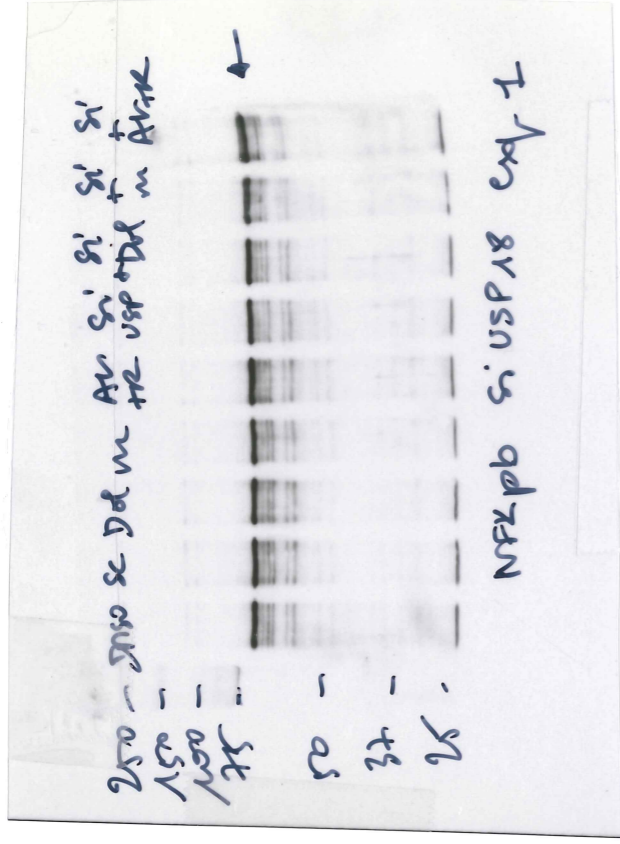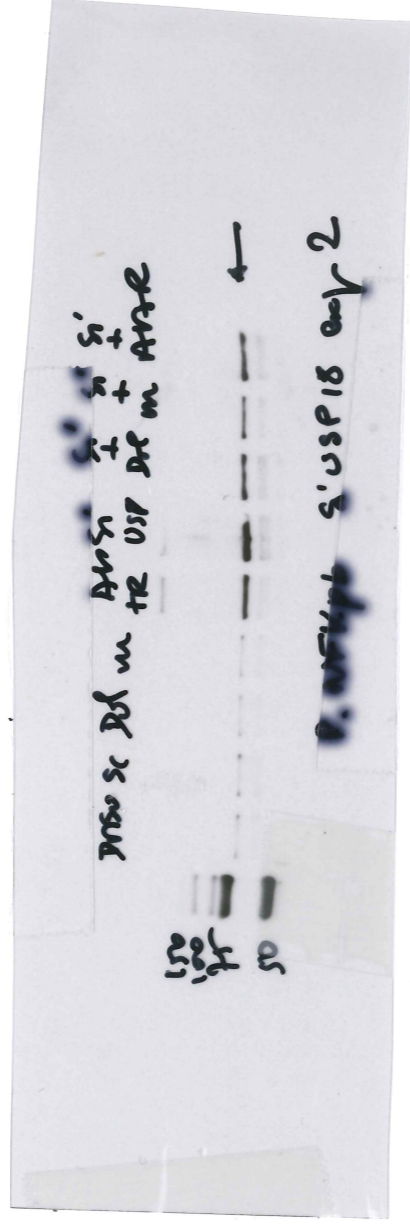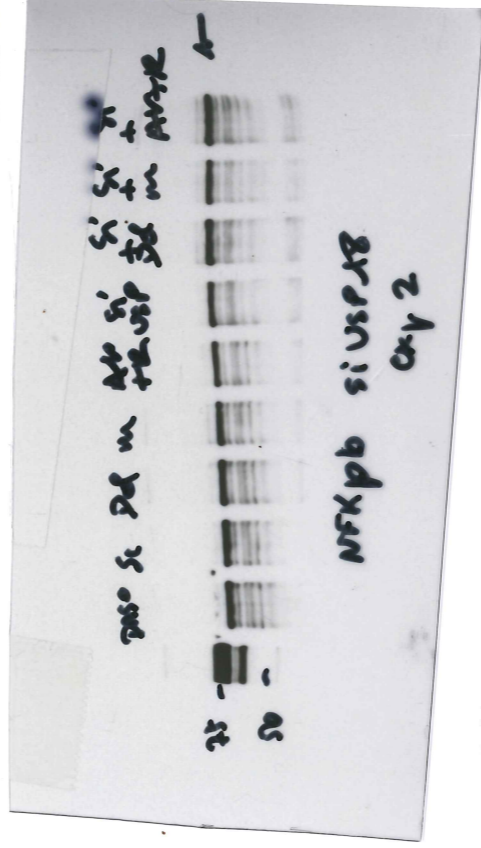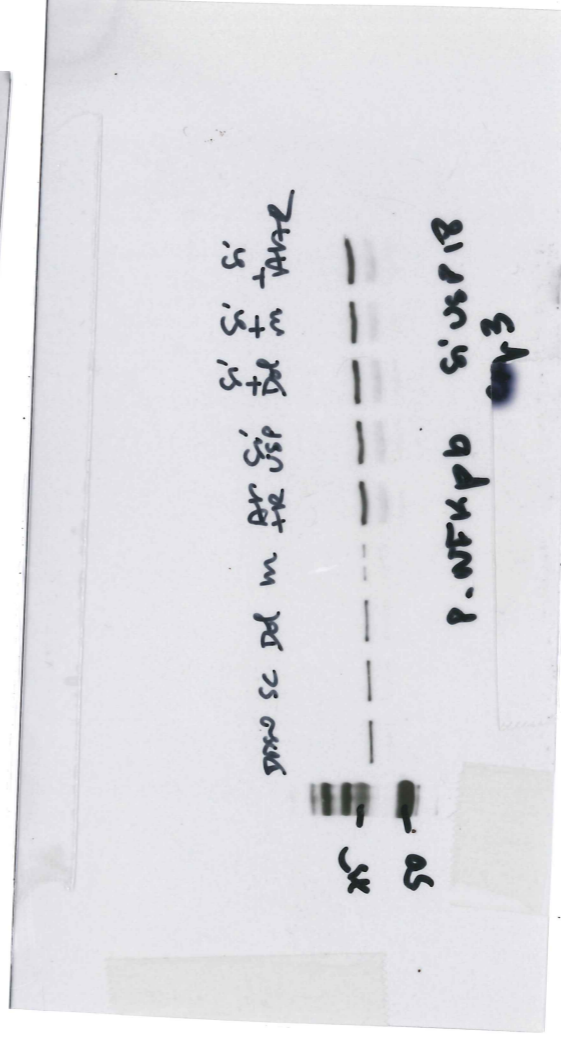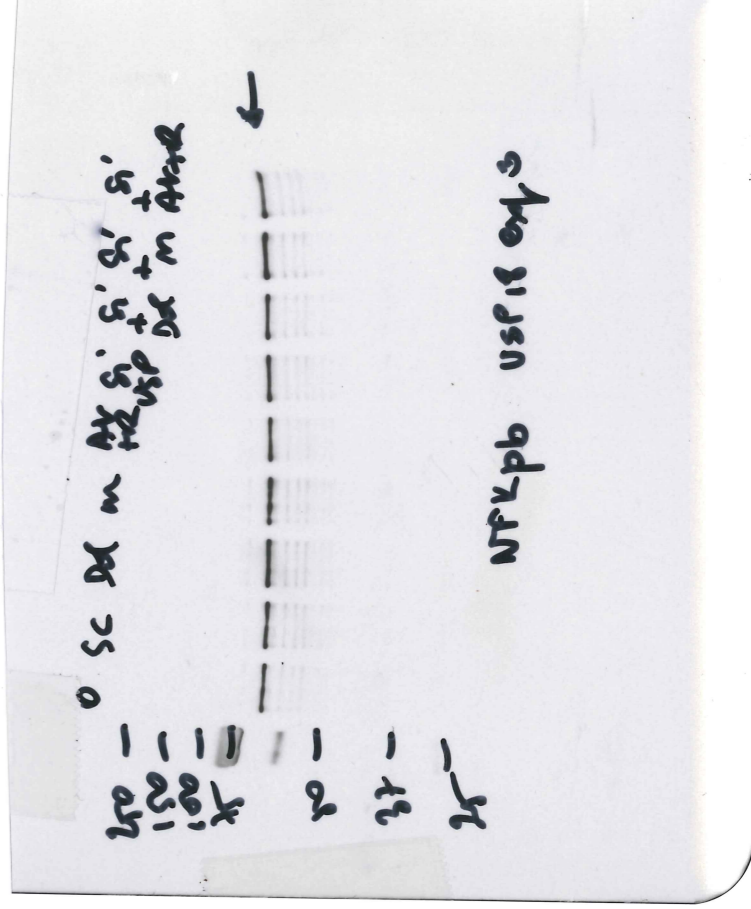

72

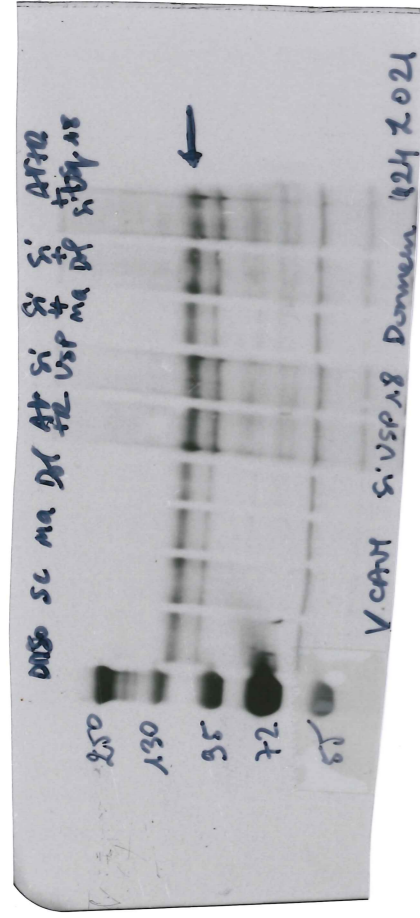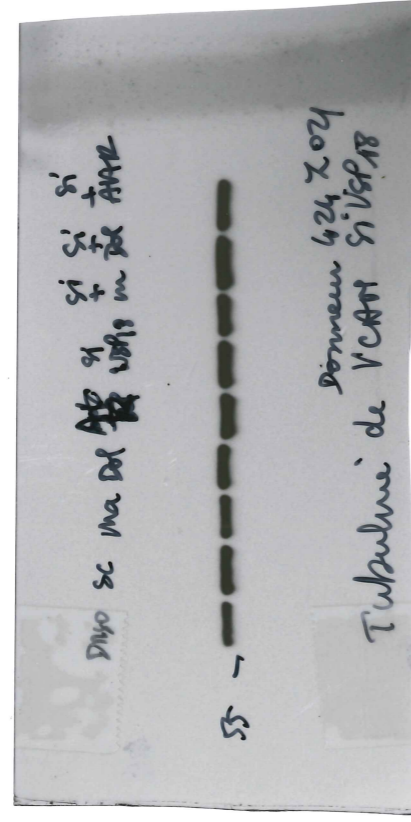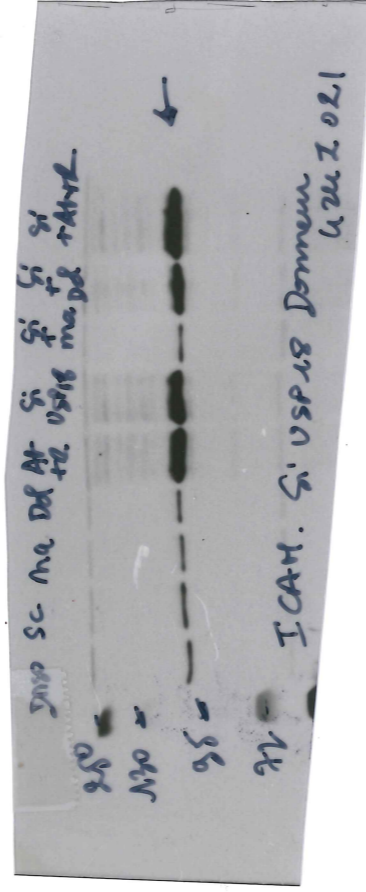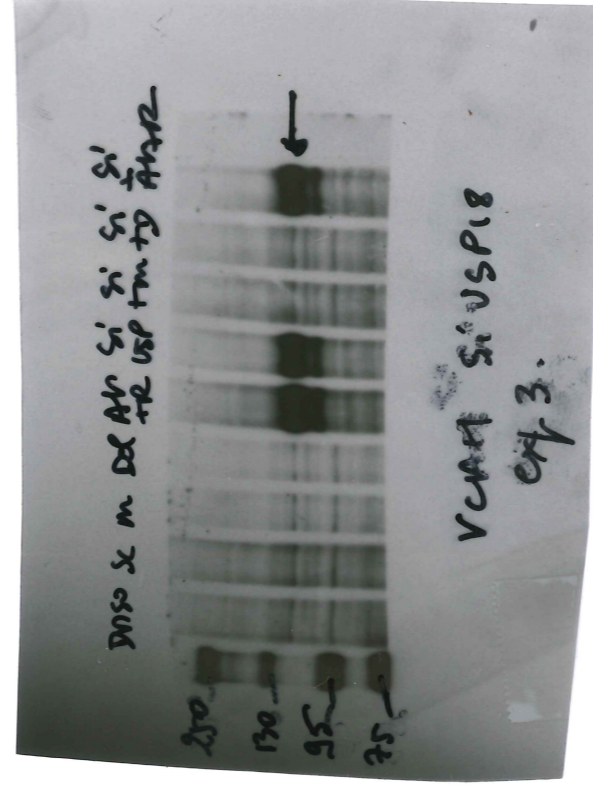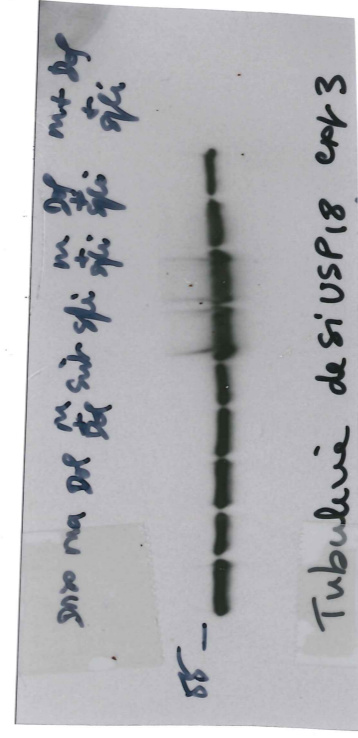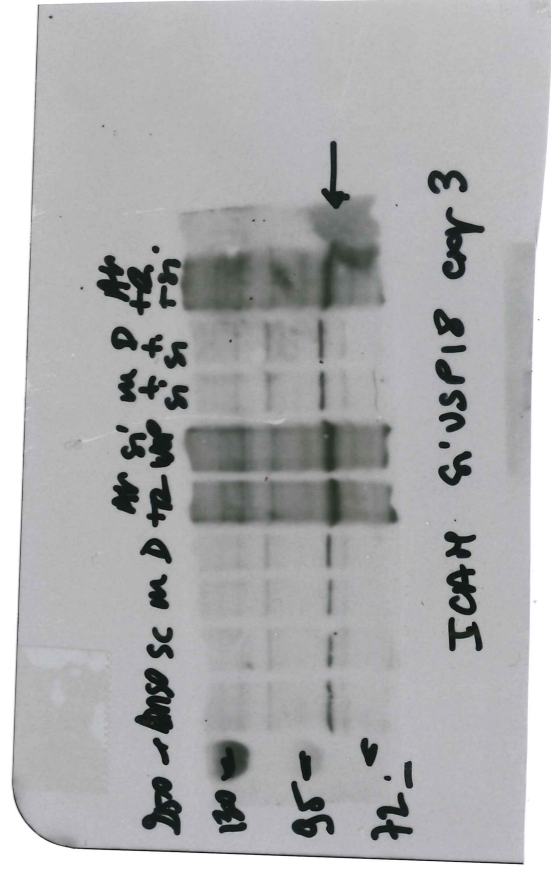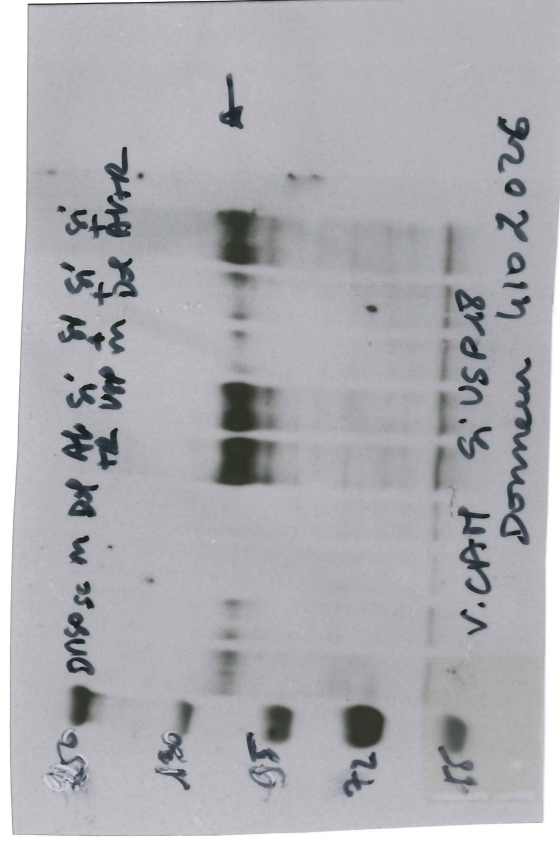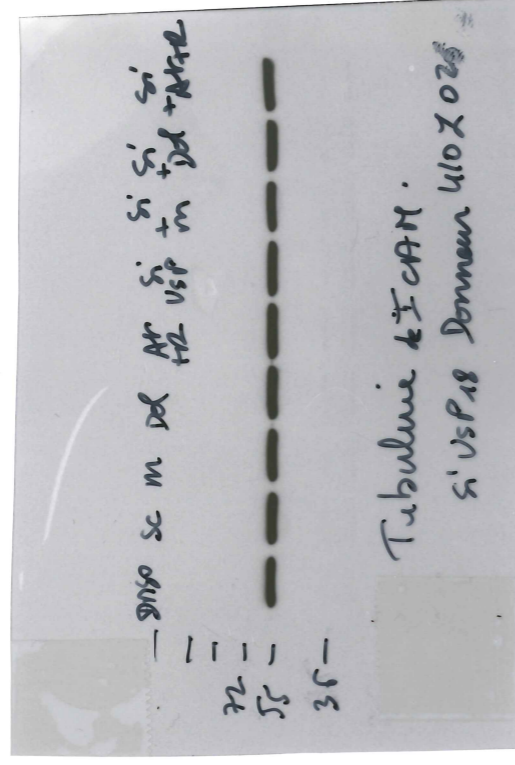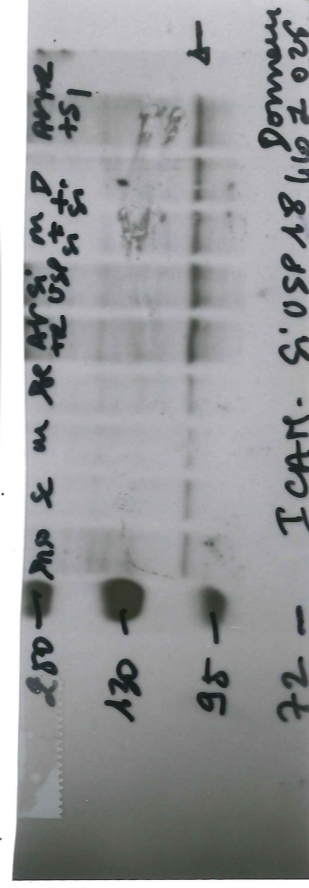

4238

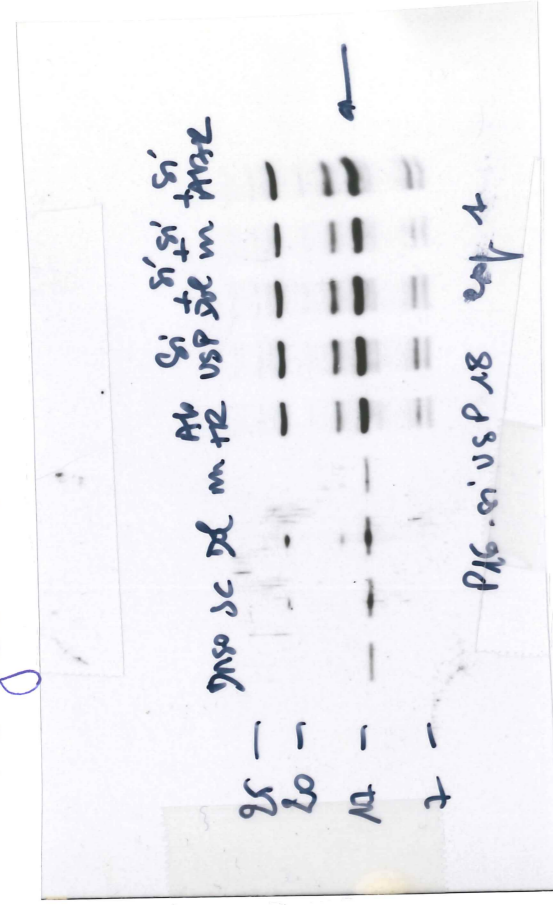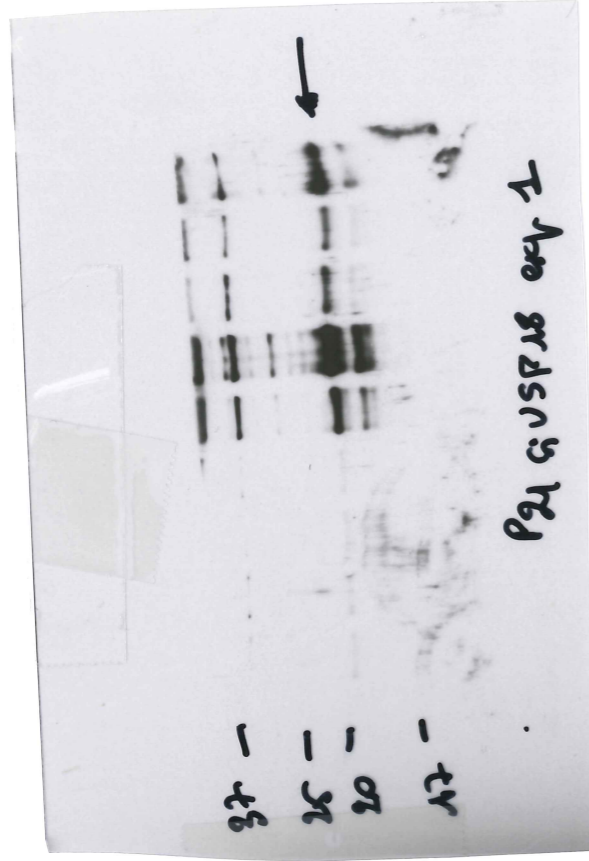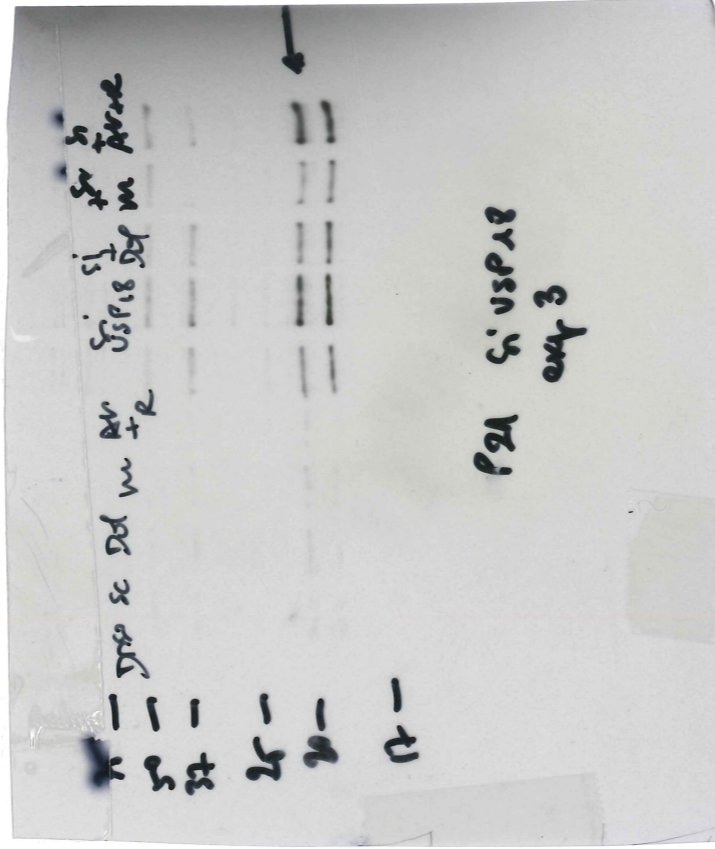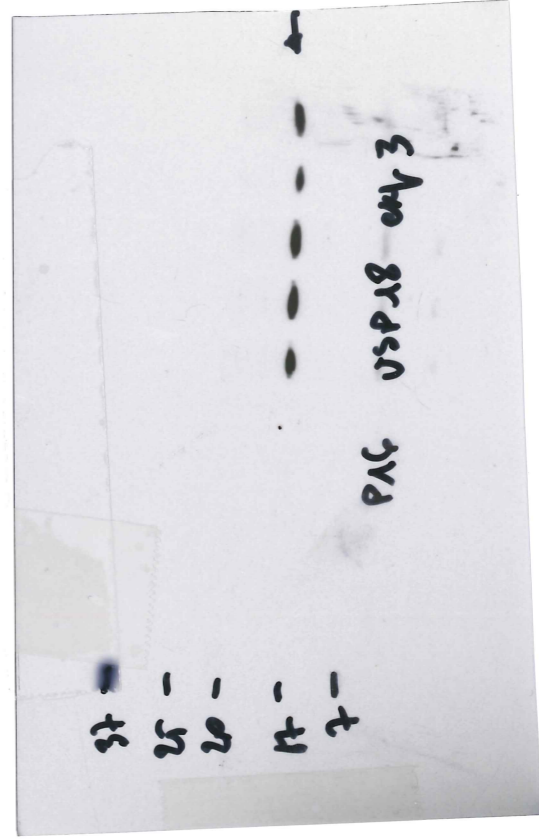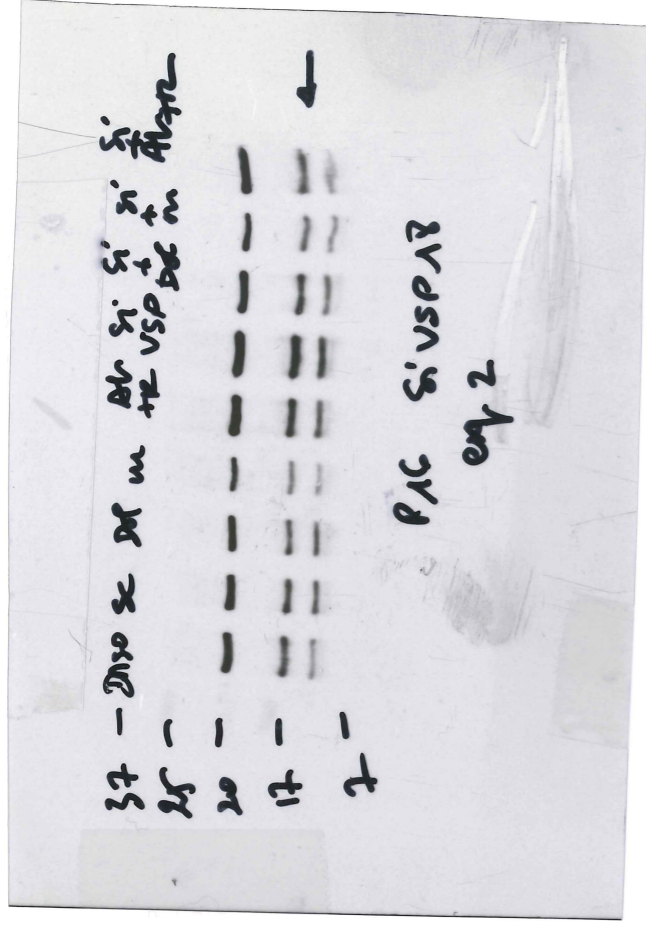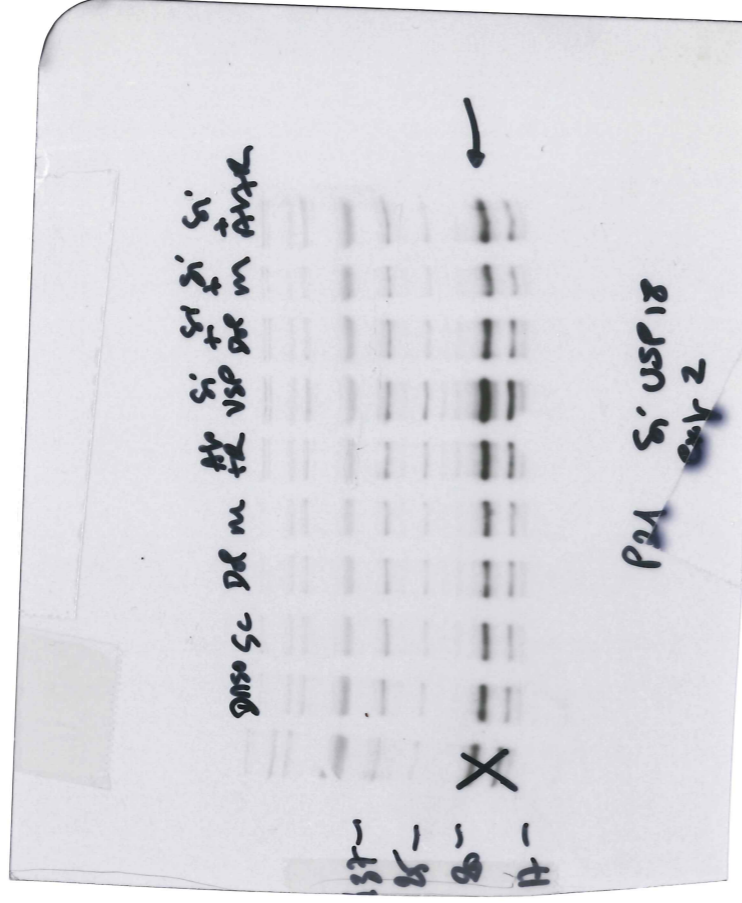

113A

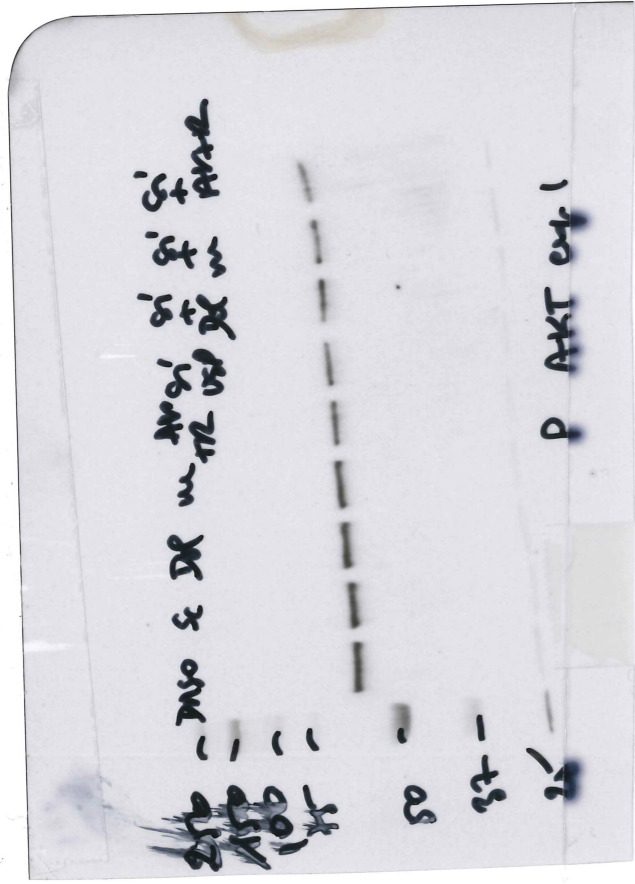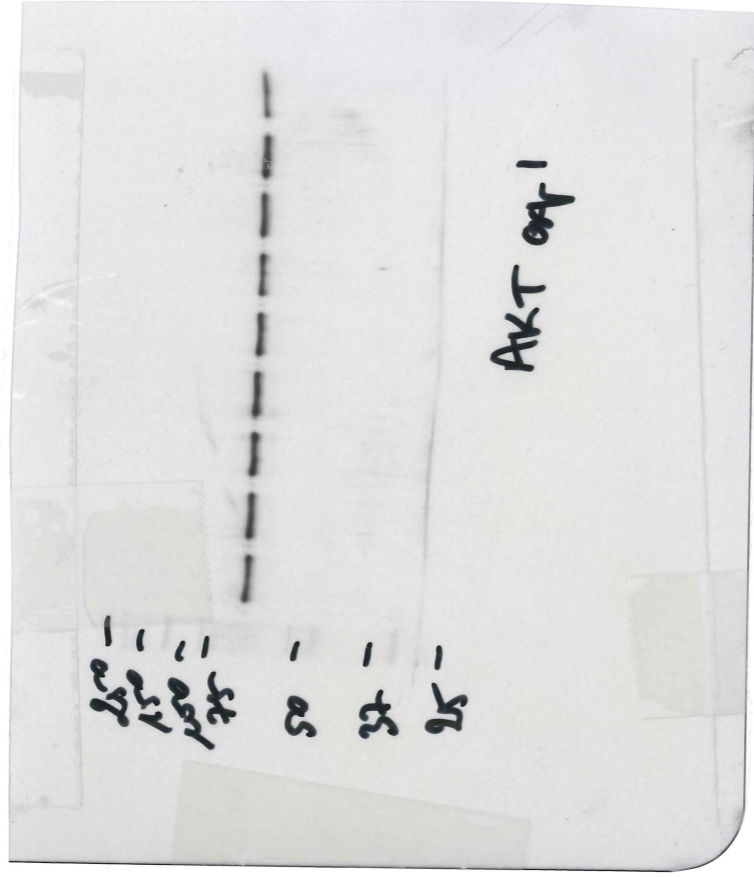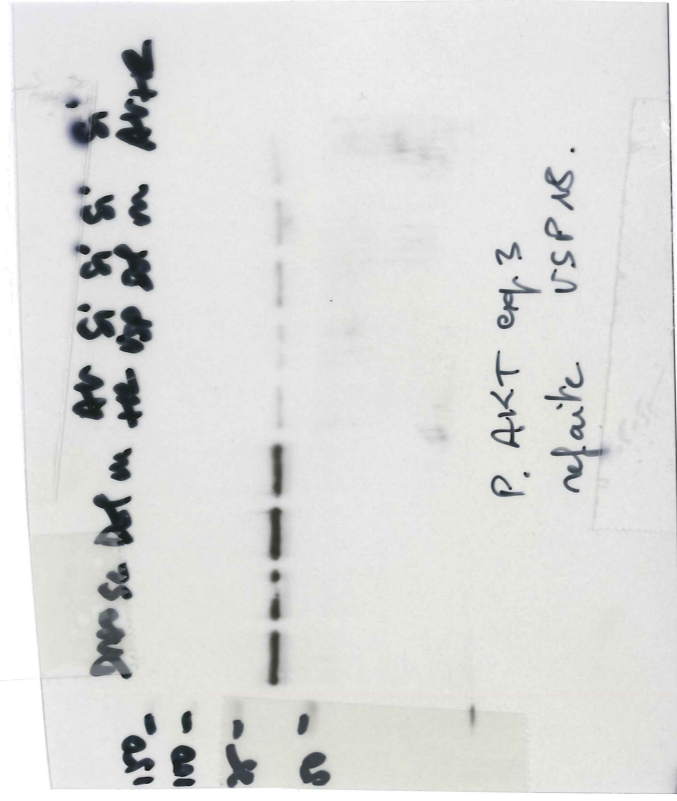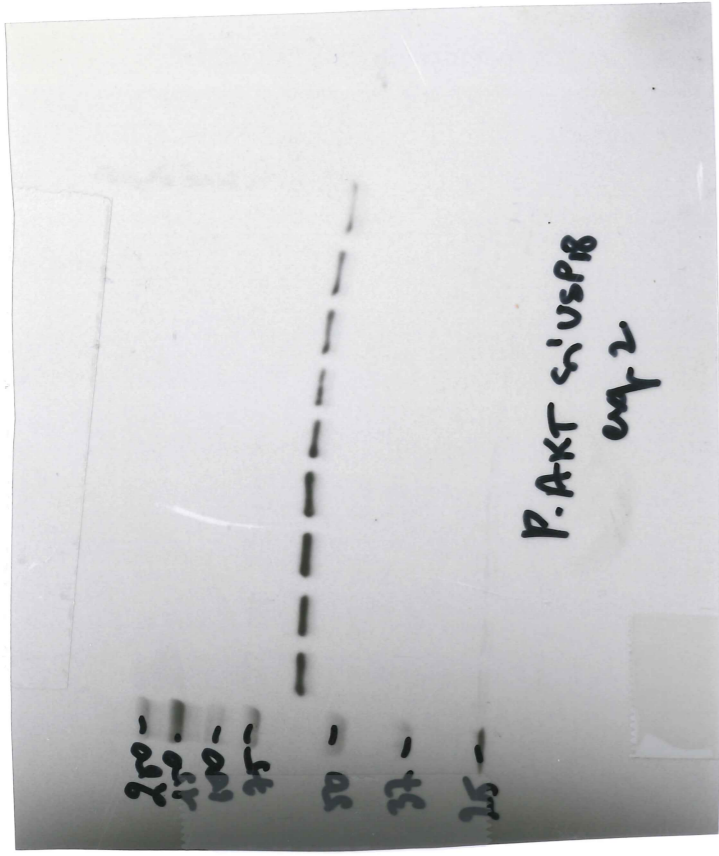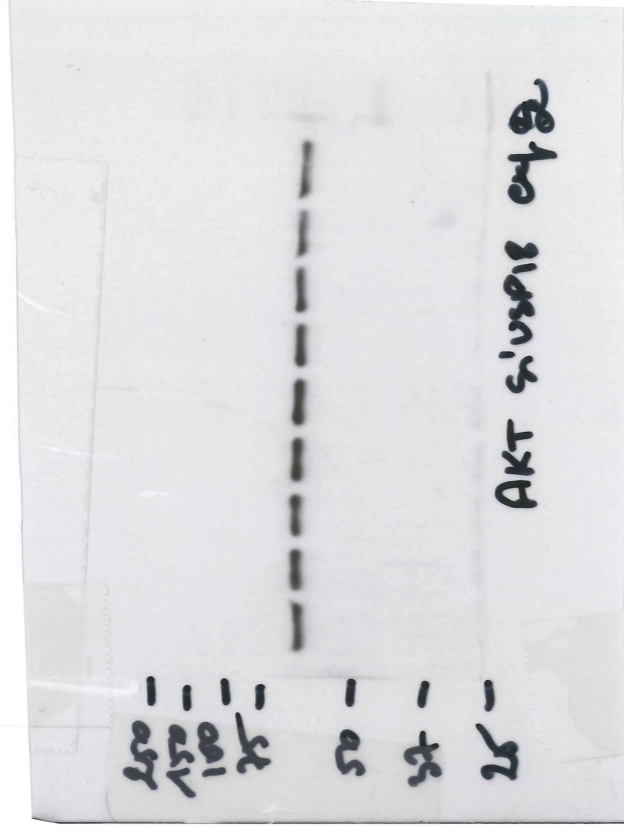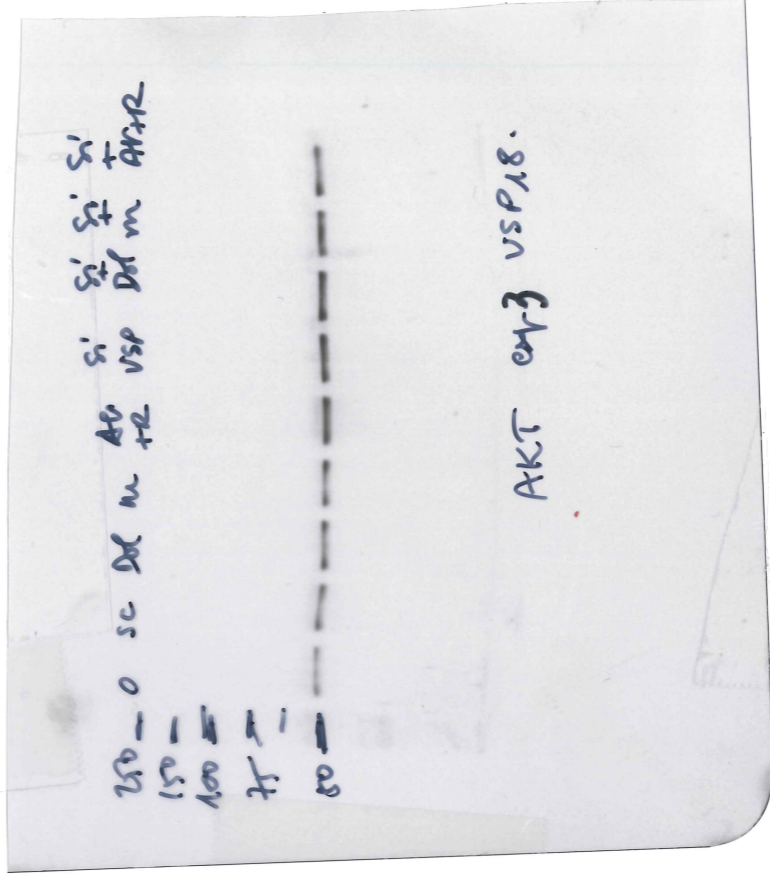

Li<sub>2</sub>FeO<sub>4</sub>

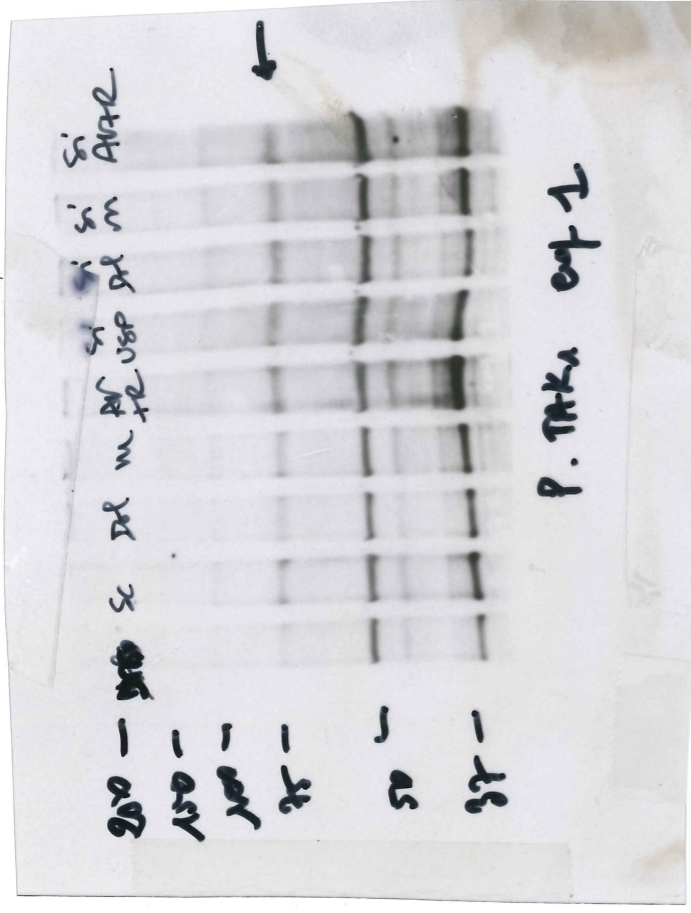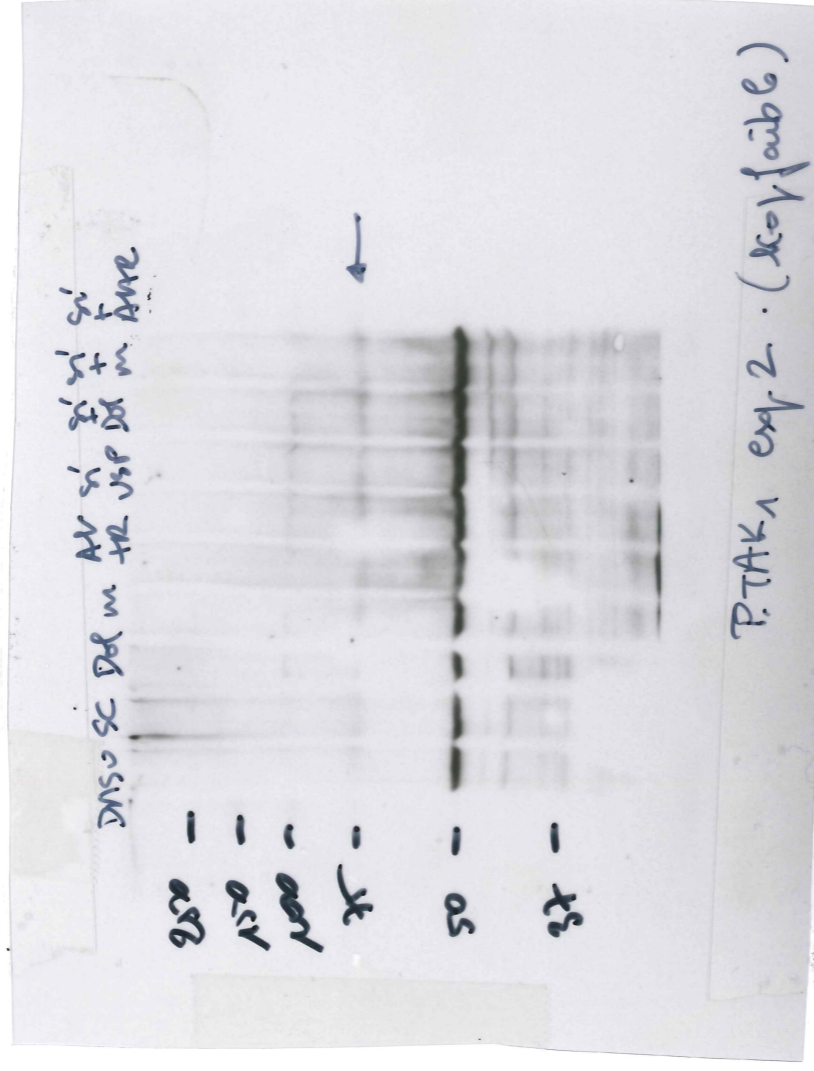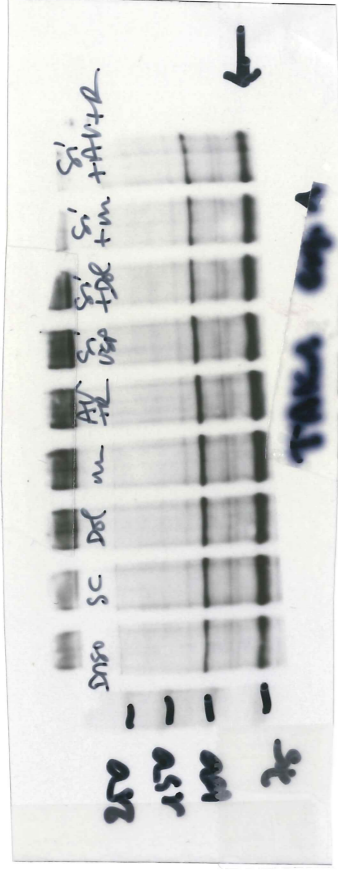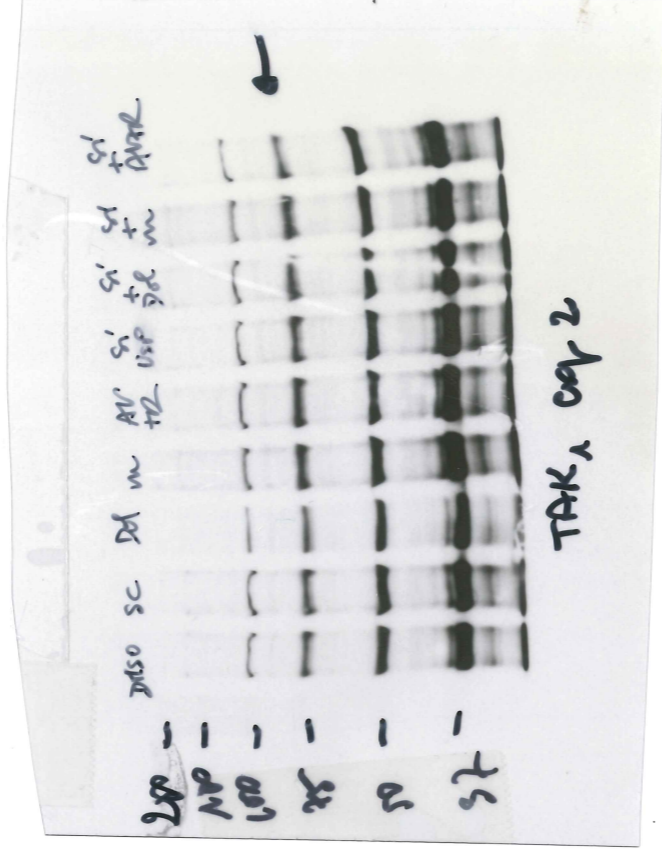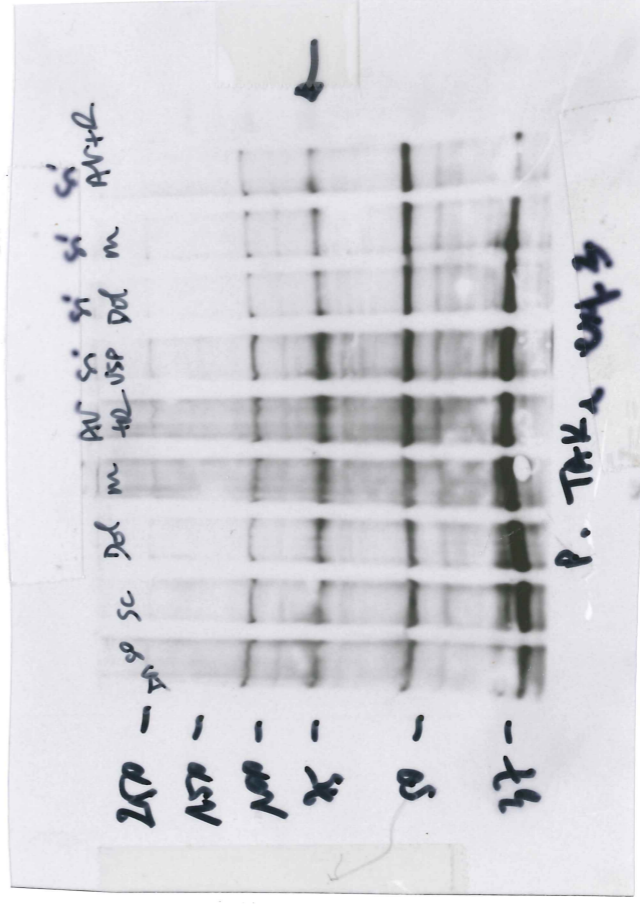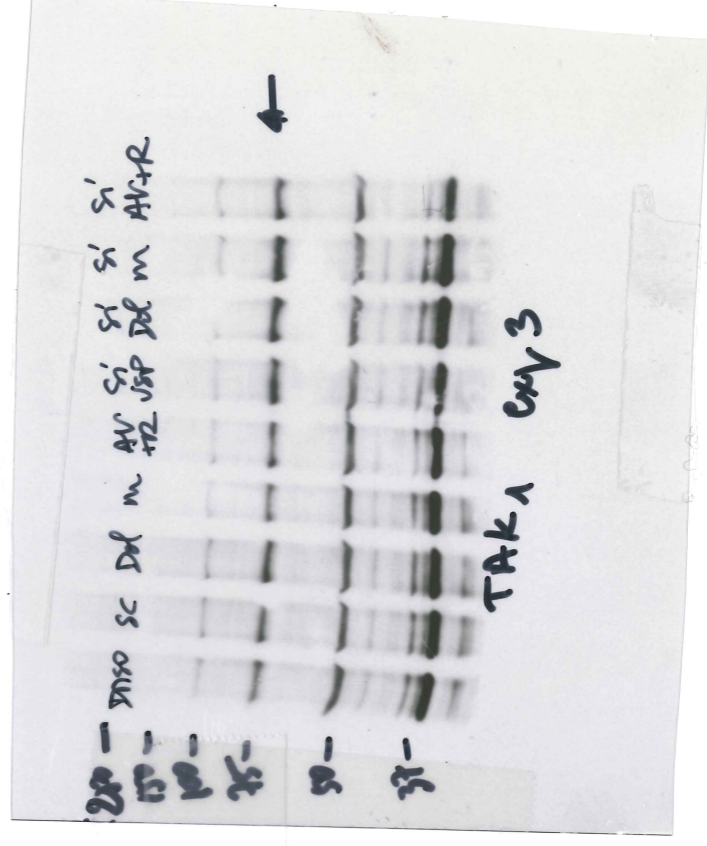

Figure 12

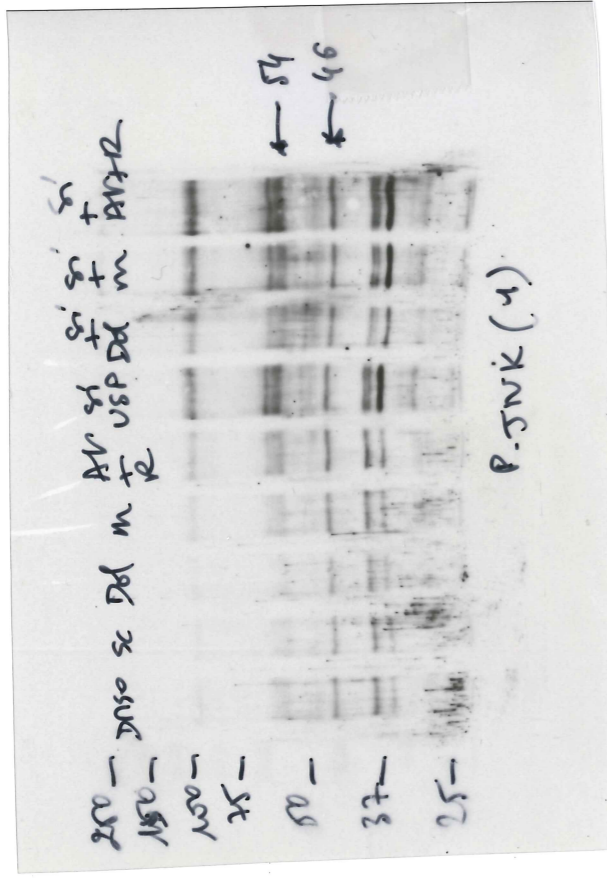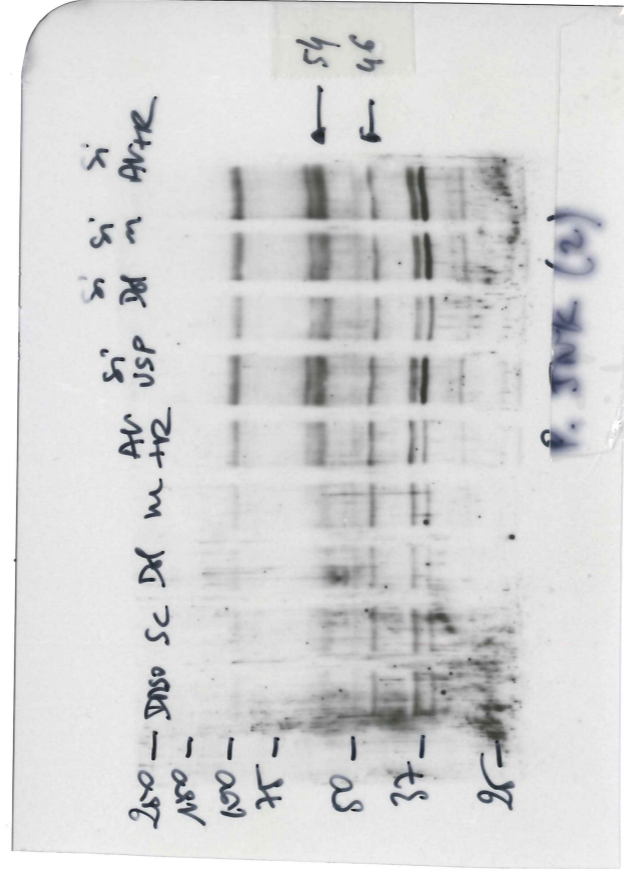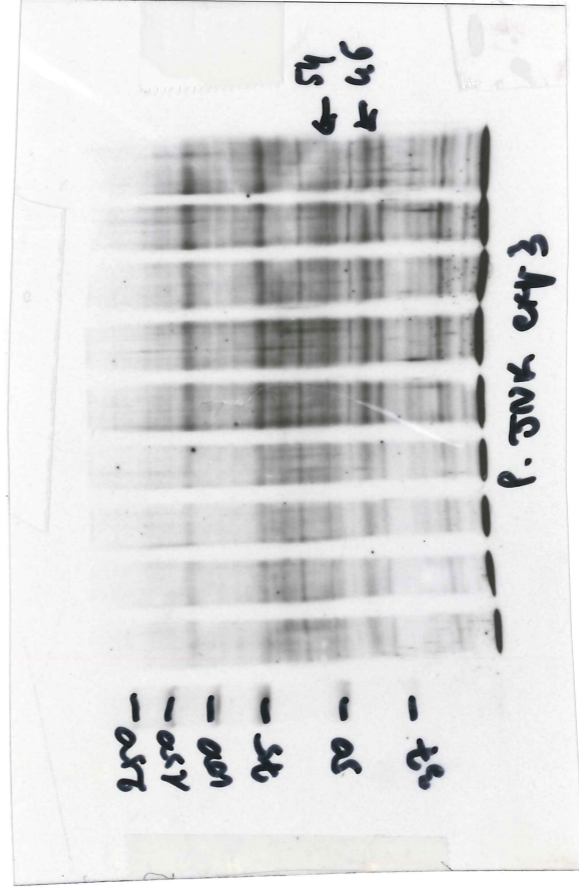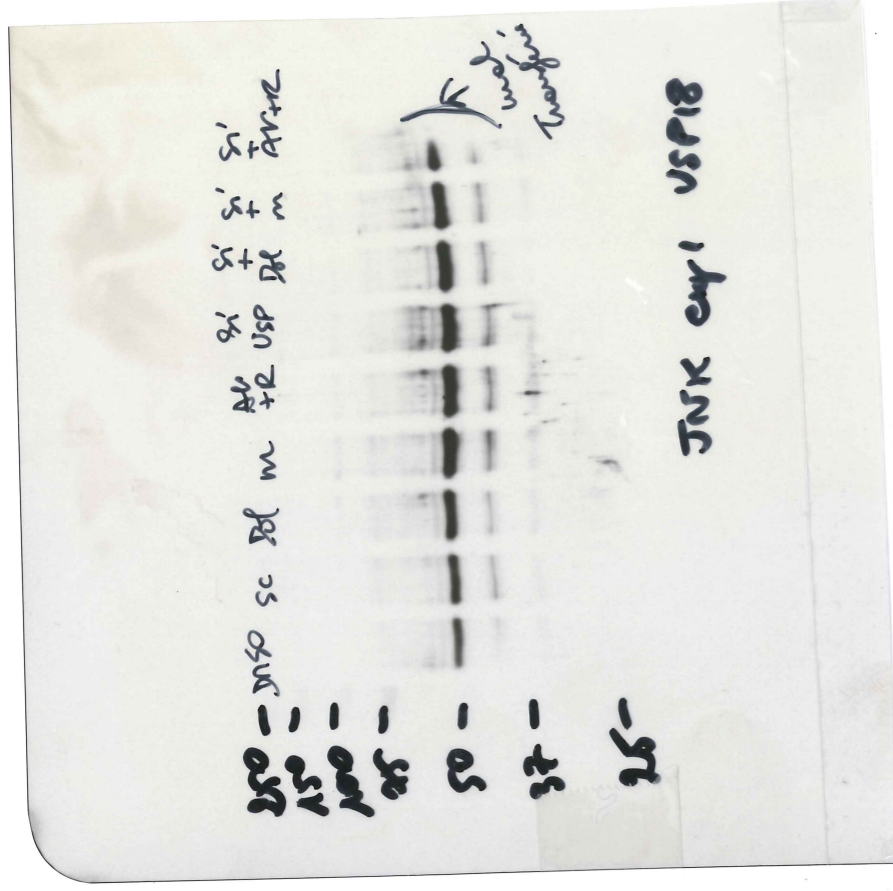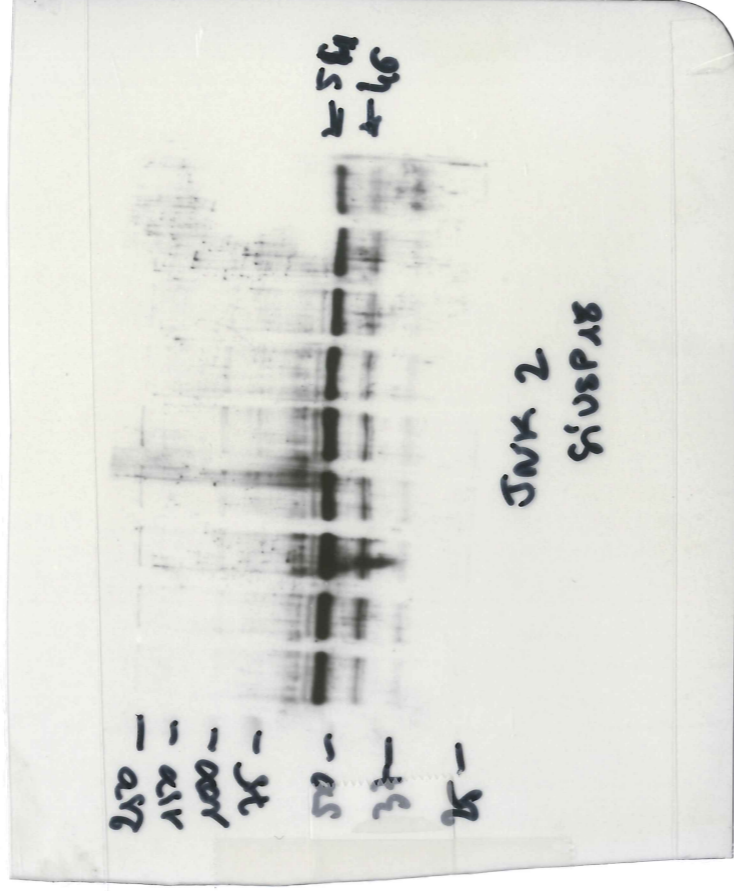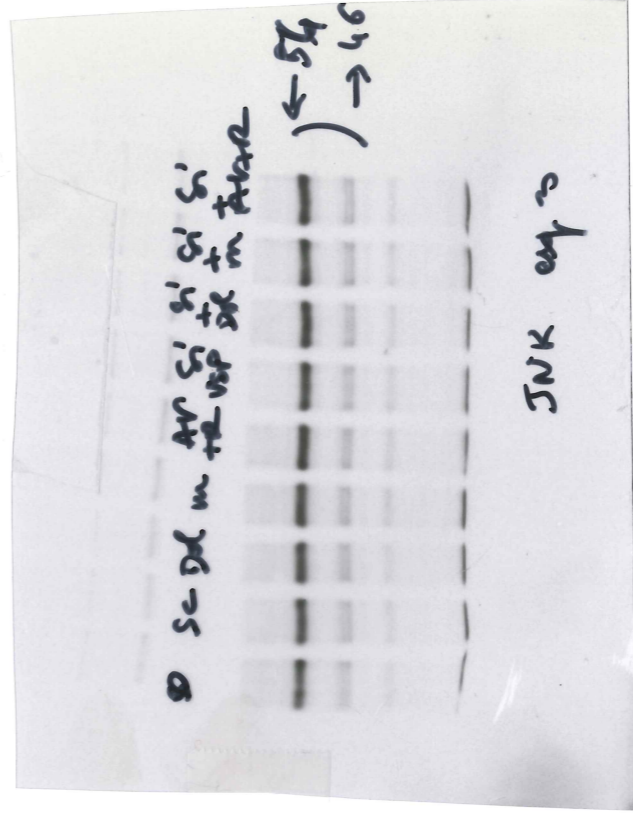

5/2/20

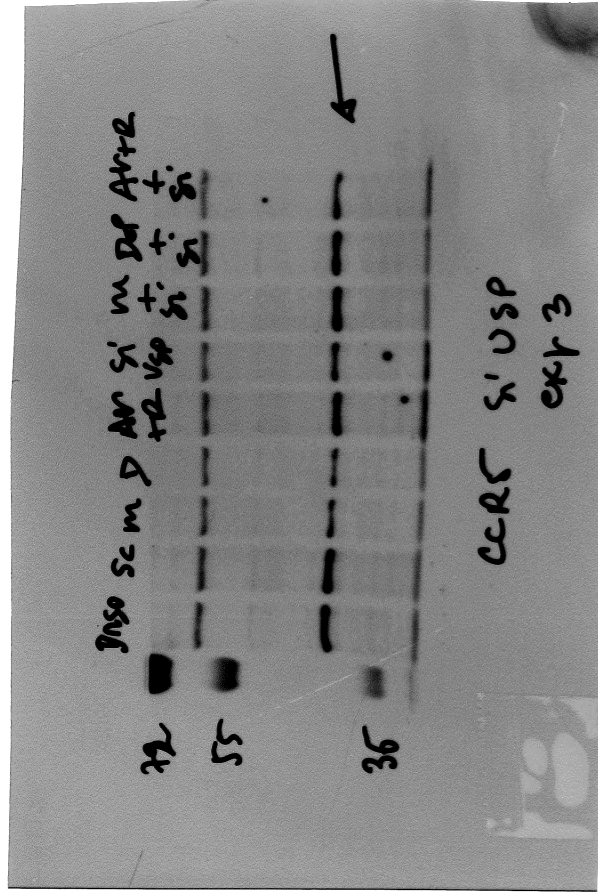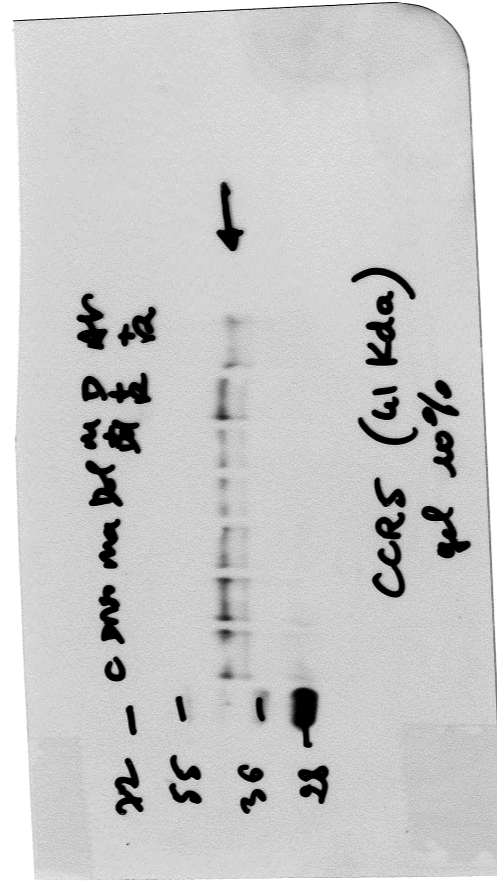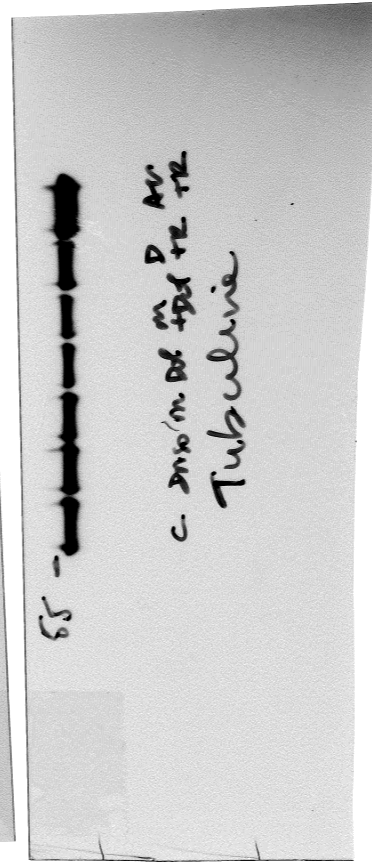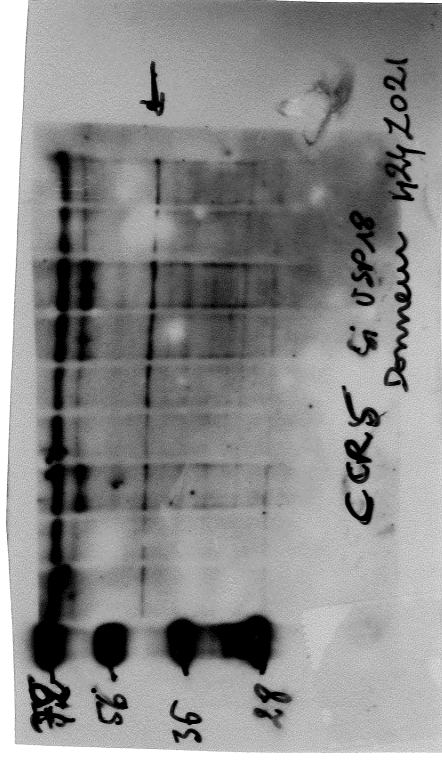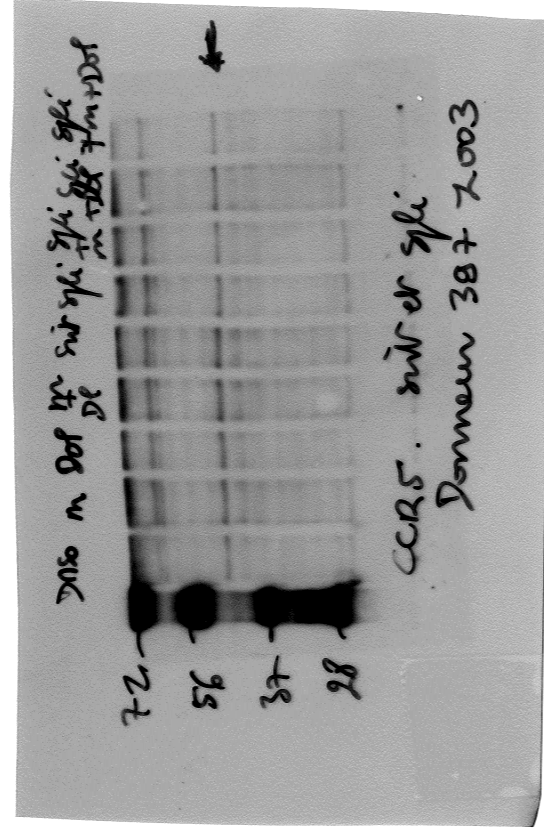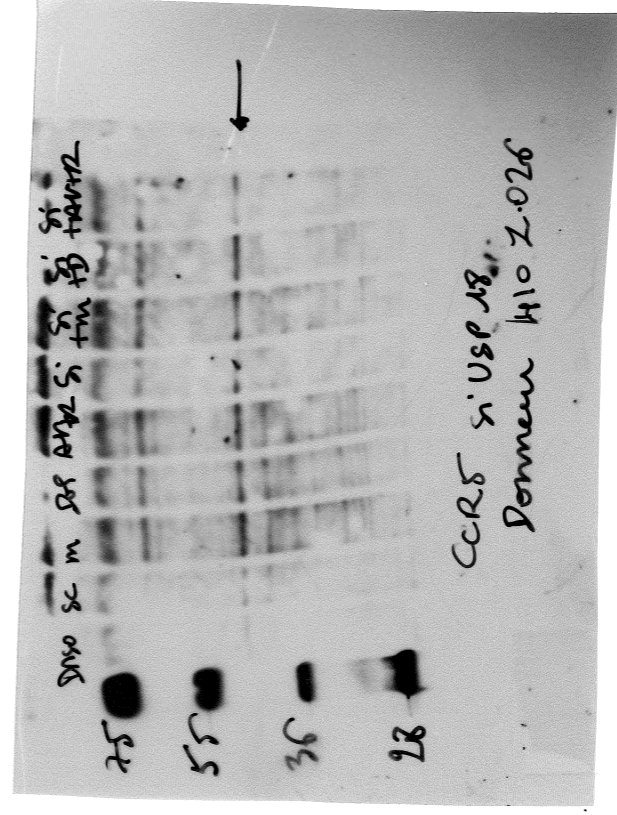

Supplement: S2 Fig — (PDF) [file pone.0226924.s002.pdf]
