# Supplementary material for: HIV antiretroviral drugs, dolutegravir, maraviroc and ritonavir-boosted atazanavir use different pathways to affect inflammation, senescence and insulin sensitivity in human coronary endothelial cells
Source: PLoS One. 2020 Jan 23;15(1):e0226924. doi: 10.1371/journal.pone.0226924 (PMC6977740; doi:10.1371/journal.pone.0226924)
Supplement: S3 Fig — (PDF) [file pone.0226924.s003.pdf]

Figure 10A: several proteins were revealed on the same blot and therefore, the blots were cut

Insulin receptor phospho-tyrosine exp 1 and 2

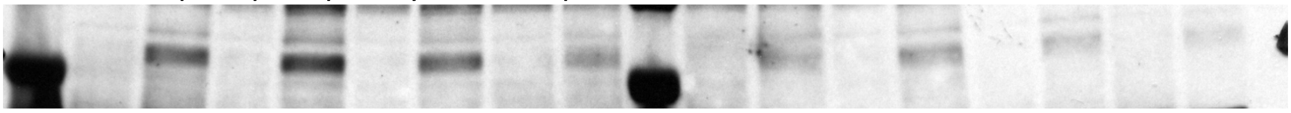

DMSO,DMSO+I,DTG, DTG+I,MVC, MVC+I, ATV/r, ATV/r+I DMSO, DMSO+I,DTG, DTG+I,MVC, MVC+IATV/r,ATV/r+I

Insulin receptor total exp 1 and 2

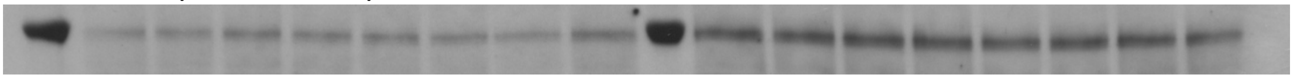

DMSO,DMSO+I,DTG, DTG+I,MVC, MVC+I, ATV/r, ATV/r+I DMSO, DMSO+I,DTG, DTG+I,MVC, MVC+IATV/r,ATV/r+I

Insulin receptor phospho-tyrosine exp 3

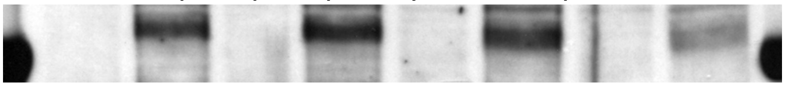

DMSO,DMSO+I,DTG, DTG+I,MVC, MVC+I, ATV/r, ATV/r+I

Insulin receptor total exp 3

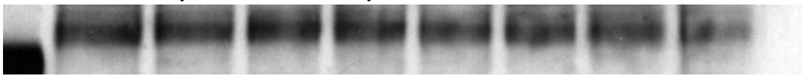

DMSO,DMSO+I,DTG, DTG+I,MVC, MVC+I, ATV/r, ATV/r+I

Figure 10B

PhosphoAKT exp 1 and 2

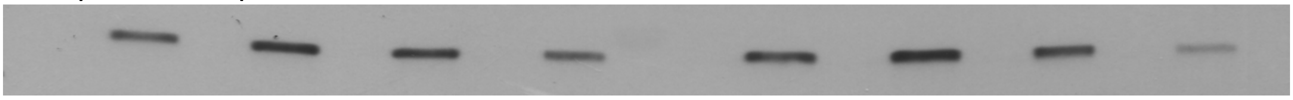

DMSO,DMSO+I,DTG, DTG+I,MVC, MVC+I, ATV/r, ATV/r+I DMSO, DMSO+I,DTG, DTG+I,MVC, MVC+IATV/r,ATV/r+I

AKT total exp1 and 2

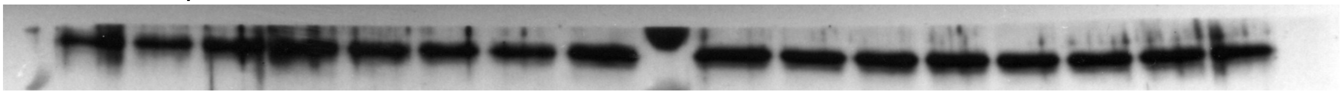

DMSO,DMSO+I,DTG, DTG+I,MVC, MVC+I, ATV/r, ATV/r+I DMSO, DMSO+I,DTG, DTG+I,MVC, MVC+IATV/r,ATV/r+I

PhosphoAKT exp 3

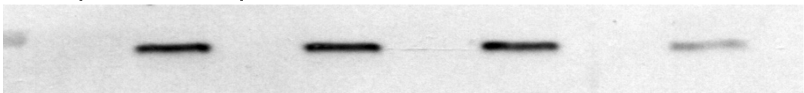

DMSO,DMSO+I,DTG, DTG+I,MVC, MVC+I, ATV/r, ATV/r+I

AKT total exp 3

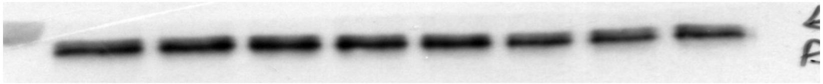

DMSO,DMSO+I,DTG, DTG+I,MVC, MVC+I, ATV/r, ATV/r+I
